# Supplementary material for: The effects of purifying selection on patterns of genetic differentiation between Drosophila melanogaster populations
Source: Heredity (Edinb). 2014 Sep 17;114(2):163–74. doi: 10.1038/hdy.2014.80 (PMC4270736; doi:10.1038/hdy.2014.80)
Supplement: Supplementary Information [file hdy201480x1.doc]

**The effects of purifying selection on patterns of genetic differentiation between *Drosophila melanogaster* populations**

Benjamin C. Jackson^1*^, José L. Campos^2^ and Kai Zeng^1*^

1. Department of Animal and Plant Sciences, University of Sheffield, Sheffield, S10 2TN, UK

2. Institute of Evolutionary Biology, School of Biological Sciences, University of Edinburgh, EH9 3JT, UK

* Corresponding authors:

Benjamin C. Jackson

e-mail: benjamincjackson@gmail.com; tel. no. 0114 222 0112

Kai Zeng

e-mail: k.zeng@sheffield.ac.uk; tel. no. 0114 222 4708

Running Title: Purifying selection and population differentiation

Word Count: 7076

**Abstract**

Using data provided by the *Drosophila* Population Genomics Project, we investigate factors that affect genetic differentiation between Rwandan and French populations of *D. melanogaster*. By examining within-population polymorphisms, we show that sites in long introns (especially those > 2000bp) have significantly lower *π* (nucleotide diversity) and more low-frequency variants (as measured by Tajima's *D*, minor allele frequencies, and prevalence of variants that are private to one of the two populations) than short introns, suggesting a positive relationship between intron length and selective constraint. A similar analysis of protein-coding polymorphisms shows that 0-fold (degenerate) sites in more conserved genes are under stronger purifying selection than those in less conserved genes. There is limited evidence that selection on codon bias has an effect on differentiation (as measured by *F_ST_*) at 4-fold (degenerate) sites, and 4-fold sites and sites in 8-30bp of short introns ≤ 65bp have comparable *F_ST_* values. Consistent with the expected effect of purifying selection, sites in long introns and 0-fold sites in conserved genes are less differentiated than those in short introns and less conserved genes, respectively. Genes in non-crossover regions (e.g., the 4th chromosome) have very high *F_ST_* values at both 0-fold and 4-fold degenerate sites, which is probably due to the large reduction in within-population diversity caused by tight linkage between many selected sites. Our analyses also reveal subtle statistical properties of *F_ST_*, which arise when information from multiple SNPs is combined and can lead to the masking of important signals of selection.

**Key words:** Population differentiation, purifying selection, introns, *F_ST_*, genetic linkage

**Introduction**

Natural populations are often divided into subpopulations. Studying the extent to which different subpopulations are genetically differentiated has been of paramount importance in evolutionary genetics, as it provides a way to examine how different evolutionary forces such as genetic drift, natural selection, and migration drive changes in the genome (reviewed in Chap. 7 of Charlesworth and Charlesworth, 2010).

Specifically, insights into fundamental processes such as historical demographic changes, (local) adaptation, and speciation can be obtained by comparing patterns of genetic differentiation across different genomic regions (Wu, 2001; Weir and Hill, 2002; Charlesworth *et al.*, 2003; Hey and Machado, 2003; Beaumont, 2005; Holsinger and Weir, 2009). For instance, by scanning for loci that show unusually high levels of differentiation relative to the rest of the genome, we can detect loci that are under diversifying selection, whereby different alleles are favoured in different subpopulations (Beaumont and Nichols, 1996; Beaumont and Balding, 2004; Foll and Gaggiotti, 2008; Excoffier *et al.*, 2009). As another example, in a study comparing African and non-African humans, it was found that the X chromosome was substantially more diverged than the autosomes, over and above the null expectation based on the fact that there are four copies of each autosome for every three copies of the X chromosome, which in turn suggests that dispersal in humans may be sex-biased or that the X chromosome may have experienced repeated selection after the divergence of African and non-African populations (Keinan *et al.*, 2009).

Genetic differentiation between subpopulations is often measured by Wright's *F_ST_* (Wright, 1951), which is abbreviated as *F* in this study. *F* can be defined as the proportion of genetic variation explained by differences in allele frequencies between subpopulations (Charlesworth, 1998; Holsinger and Weir, 2009; Bhatia *et al.*, 2013). *F* ranges between 0 and 1, which indicate no differentiation and fixed differences between subpopulations, respectively. Various genetic data, e.g. single nucleotide polymorphisms (SNPs) and microsatellites, can be used to estimate *F*, but using statistical procedures that take into account biological properties of the data under consideration (e.g., high versus low mutation rate) is vital for acquiring accurate estimates (Weir and Cockerham, 1984; Excoffier *et al.*, 1992; Slatkin, 1995; Nagylaki, 1998; Holsinger and Weir, 2009).

*Drosophila melanogaster*, a classic model organism for population genetics, offers an invaluable system for studying population differentiation. Despite having a worldwide distribution in the present day, it is believed that the species originated in sub-Saharan Africa (David and Capy, 1988; Stephan and Li, 2007). The colonisation of Europe has been suggested to have taken place about fifteen thousand years ago (David and Capy, 1988; Stephan and Li, 2007; Duchen *et al.*, 2013). The Americas and Australia were colonised much more recently, possibly in the past few hundred years (David and Capy, 1988; Stephan and Li, 2007; Duchen *et al*, 2013). By studying patterns of genetic differentiation, investigators have obtained evidence that American populations of *D. melanogaster* may be formed by admixture between African and European flies (Caracristi and Schlotterer, 2003). Multiple attempts have also been made to identify loci with unusually high *F,* which may have contributed to local adaptation to different habitats (Turner *et al.*, 2008; Yukilevich *et al.*, 2010; Kolaczkowski *et al.*, 2011; Fabian *et al.*, 2012; Langley *et al.*, 2012; Pool *et al.*, 2012; Campo *et al.*, 2013).

These previous studies of *D. melanogaster* have mainly focused on determining the evolutionary relationship between subpopulations, quantifying the overall level of differentiation, and detecting genomic regions of interest using outlier scans. However, the role of purifying selection in shaping large-scale patterns of differentiation has not been well characterised, although it has been widely accepted that the majority of new mutations that affect fitness will have detrimental effects (Pal *et al.*, 2006; Eyre-Walker and Keightley, 2007). Supporting this view, it has been estimated that only between 1% and 2% of new nonsynonymous mutations in *D. melanogaster* are (weakly) positively selected, and about 6% are nearly neutral (i.e., |*N_e_s*| ≤ 1, where *N_e_* is the effective population size and *s* the selection coefficient), and the remaining are deleterious (|*N_e_s*| > 1) (Keightley and Eyre-Walker, 2007; Eyre-Walker and Keightley, 2009; Schneider *et al.*, 2011). Thus, we are interested in testing the following predictions based on population genetic theory of subdivided populations (reviewed in Chap. 7 of Charlesworth and Charlesworth, 2010): (*i*) purifying selection reduces differentiation between populations at functionally important regions; (*ii*) the level of reduction is positively correlated with the level of selective constraint. Answering these questions will help us better understand the sources of variation in genetic differentiation across the genome, which is important for example, in interpreting results obtained from genome scans (Beaumont and Nichols, 1996; Beaumont and Balding, 2004; Foll and Gaggiotti, 2008; Excoffier *et al*, 2009).

We will address the questions raised above by making use of the high-quality whole-genome resequencing data published by the *Drosophila* Population Genomics Project (DPGP) for one French population and one Rwandan population (Langley *et al*, 2012; Pool *et al*, 2012). In addition to protein-coding regions, we investigate introns, as previous studies have shown strong evidence that these genomic regions are under substantial selective constraints, probably as a result of the presence of *cis*-regulatory elements and noncoding RNA genes (Bergman and Kreitman, 2001; Parsch, 2003; Andolfatto, 2005; Haddrill *et al.*, 2005a; Halligan and Keightley, 2006; Casillas *et al.*, 2007; Roy *et al.*, 2010).

Our study proceeds as follows. First, we present an overview of patterns of genetic variation both within and between populations using data from genomic regions where crossing over occurs (crossover regions). We are interested in understanding whether patterns of differentiation at 4-fold degenerate (hereafter 4-fold) sites are affected by selection on codon usage, and whether 4-fold sites and putatively neutral sites in 8-30 bp regions of introns ≤ 65 bp (Halligan and Keightley, 2006; Parsch *et al.*, 2010) are comparable with respect to levels of differentiation. These are intended to identify putatively neutral sites which can be used as a reference in the study of the effects of purifying selection on genetic differentiation. We then examine the relationship between *K_A_* (nonsynonymous divergence) and diversity/differentiation patterns at 0-fold degenerate (hereafter 0-fold) sites in protein-coding regions, as well as the relationship between intron length and diversity/differentiation patterns in intronic regions. Finally, we compare non-crossover regions (e.g., the fourth chromosome) and crossover regions regarding differentiation patterns, study the relative contribution of selection and genetic linkage, and examine the correlation between local recombination rates and *F* at putatively neutral sites.

**Materials and Methods**

*Data acquisition*

To obtain polymorphism and divergence data we downloaded FASTQ files from the *Drosophila* Population Genomics Project (http://www.dpgp.org/dpgp2/candidate/) for 17 Rwandan *D. melanogaster* samples (RG18N, RG19, RG2, RG22, RG24, RG25, RG28, RG3, RG32N, RG33, RG34, RG36, RG38N, RG4N, RG5, RG7 and RG9), which have been estimated to have the lowest estimated levels of admixture with European populations (less than 3%, see Figure 3B of Pool *et al*, 2012). We also selected seven samples from the French population (FR14, FR151, FR180, FR207, FR217, FR310 and FR361). We will refer to these two samples as RG and FR, respectively. We further masked any regions of the African samples with evidence of admixture from European populations, using the admixture coordinates reported by Pool et al. (2012). Sites with a quality score below 31 (equivalent to a PHRED score of 48, and approximately equivalent to one error per 100kb; see Pool et al. 2012) were also masked.

From the FASTQ files we extracted protein-coding regions in crossover regions, which we abbreviate as CDS-C, using gene annotations from FlyBase release version 5.44 (www.flybase.org) and made FASTA files containing all samples (24 alleles). For each *D. melanogaster* gene with multiple transcripts, we chose one transcript randomly.

Introns belonging to our chosen transcript were extracted, and were further processed by masking any coding regions that form part of the other transcripts. Only introns occurring in crossover regions were retained (polymorphism data for introns in non-crossover regions were of significantly lower quality, and were therefore excluded).

Protein-coding regions in non-crossover regions of the *D. melanogaster* genome, abbreviated as CDS-NC, were obtained from Campos et al (2012). These data included five unlinked regions: N2 (genes located in heterochromatic regions near the centromere of the second chromosome), N3 (genes located in heterochromatic regions near the centromere of the third chromosome), N4 (the fourth chromosome), NXc (X-linked genes near the centromere), and NXt (X-linked genes near the telomere).

For all CDS-C and CDS-NC we selected *D.* *yakuba* as an outgroup to avoid any major influence of ancestral polymorphisms on the estimation of sequence divergence, which can potentially create spurious correlations between sequence divergence and recombination (e.g., Cutter and Choi 2010). One-to-one orthologous *D.* *yakuba* sequences were obtained from FlyBase (available at ftp://ftp.flybase.net/releases/FB2012_02/precomputed_files/genes/gene_orthologs_fb_2012_02.tsv.gz). We then performed amino-acid sequence alignments using MAFFT (Katoh *et al.*, 2002). These amino-acid sequence alignments were translated back to nucleotides using custom scripts in PERL to produce in-frame coding sequence alignments that included the 24 *D. melanogaster* alleles and the *D. yakuba* reference.

For introns we used *D. simulans* as an outgroup since we considered *D. yakuba* too distant for producing reliable alignments, due to the increased prevalence of indels in non-coding regions. We obtained orthologous intronic *D. simulans* sequences from Hu et al. (2013), which was based on an updated *D. simulans* genome assembly and careful alignment procedures to preserve gene structures (<http://genomics.princeton.edu/AndolfattoLab/w501_genome_files/alnMSY.tar.gz>).

Recombination rate for the midpoint of all alignments was obtained using the *Drosophila melanogaster* Recombination Rate Calculator v2.3 (Fiston-Lavier *et al.*, 2010) and the high-resolution genetic map published recently by Comeron et al. (2012).

*Sequence Analysis*

For CDS-C and CDS-NC, we calculated *K_A_* and *K_S_* (the numbers of nonsynonymous and synonymous substitutions per nonsynonymous and synonymous site, respectively) using the kaks() function from the seqinr package (Charif and Lobry, 2007) in R (http://www.r-project.org/), which implements the method of Li (1993). For introns, we calculated divergence (*K*) to the *D. simulans* reference using the dist.dna() function in the ape package of R (Paradis *et al.*, 2004), with the ‘K80’ method (Kimura, 1980). For conducting analyses using polymorphism data in the two *D. melanogaster* samples, we split CDS-C and CDS-NC into 0-fold degenerate sites and 4-fold degenerate sites by analysing the alignments codon by codon. A codon column was retained if the following requirements were met: (*i*) data from all individuals were available; (*ii*) it had at most one SNP. These were to avoid uncertainty of the order of mutations in codons with multiple SNPs. We retained 7235 autosomal and 1150 X-linked CDS alignments for which we had both more than 10 bp of 0-fold sites and more than 10bp of 4-fold sites.

We split introns into short (≤ 65bp long) and long (> 65bp long) classes, and further trimmed short introns to retain positions 8-30 from the 5’ end (the SI sites), in order to retain sites under the least amount of selective constraint (Halligan and Keightley, 2006; Parsch *et al*, 2010). This left us with 7483 autosomal and 752 X-linked short introns, and 8851 autosomal and 1869 X-linked long introns. To keep the sample size the same as the CDS data, only intronic sites where data from all individuals were available were retained. This requirement appears to be conservative with respect to detecting SNPs (note also that regions within 3 bp of an indel were also masked by DPGP; see Pool et al. 2012 and http://www.dpgp.org/dpgp2/DPGP2.html). For instance, *π* (nucleotide diversity) estimated using data from SI sites that fulfilled the above criterion was 0.0146 in the RG sample, whereas the estimate increased to 0.0164 when we retained, within the same genomic regions, all sites that had data from at least two individuals. However, as we will show in the Results, there is no detectable difference between the SI and 4-fold sites in terms of skewness of allele frequency spectrum (as measured by Tajima's *D*), average minor allele frequency, and *F_ST_*. Our conservative data filtering procedure is unlikely to bias our analysis of population differentiation.

Because of the complete linkage between the CDS-NC genes located within a non-crossover (NC) region, each non-recombining region of the genome effectively represents a single locus. Therefore all genes within a single NC region were concatenated and analysed as a single gene. In total, we have three autosomal NC regions, which are N2, N3, and N4, and two X-linked NC regions, which are NXc and NXt. These loci were kept intact in the permutation tests used to compare values of summary statistics calculated using data from NC and crossover (C) regions.

Nucleotide diversity (*π*), Tajima’s *D* (Tajima, 1989), and relative Tajima’s *D*, (Schaeffer, 2002) were calculated using nuc.div() and a modified version of the tajima.test(), both from the pegas package in R (Paradis, 2010). Since conclusions drawn from Tajima's *D* and relative Tajima's *D* are identical, only the former is presented. Permutation tests were carried out to assess whether these statistics were different between different types of sites. For instance, to compare whether values of Tajima's *D* at 4-fold sites and SI sites were comparable, 10,000 pseudosamples were generated by randomly shuffling both the 4-fold and SI sites in the data (SNPs from a single locus were shuffled as a unit), such that in each pseudosample there were similar numbers of SNPs in the two "site classes" as in the real data.

To assess the effects of selection on codon bias on population differentiation at 4-fold sites, we calculated Fop (frequency of optimal codons) with CodonW (Peden, 1999), using the built-in table of optimal codons for *D. melanogaster*.

*Measuring population differentiation*

Levels of differentiation between the two populations of *D. melanogaster* were measured by Wright's *F_ST_*, which is abbreviated as *F*. We used the definition of Weir and Cockerham (1984), which can be expressed as

$$F=\frac{\pi_{B}-\pi_{S}}{\pi_{B}}$$

where *π_B_* is the expected divergence between a pair of alleles sampled from two different populations, and *π_S_* is the expected within-population diversity (see also Charlesworth, 1998; Keinan *et al.*, 2007). Previous investigations (Maruki *et al.*, 2012; Jakobsson *et al.*, 2013) of the dependence of *F* on minor allele frequency (MAF) were based on a different definition of *F* put forward by Nei (1973). We therefore derive the maximum value of Weir and Cockerham's definition of *F* as a function of MAF.

Consider a population divided into two subpopulations. We examine a biallelic locus. The frequency of one of the two alleles in the *k*-th subpopulation is referred to as *p_k_­* (*k* = 1 or 2). It can be shown that *π_B_* = *p*_1_(1 – *p*_2_) + *p*_2_(1 – *p*_1_) and *π_S_* = *p*_1_(1 – *p*_1_) + *p*_2_(1 – *p*_2_) (Charlesworth, 1998). Substituting these into Eq. (1), *F* can be rewritten as

$$F=\frac{2\delta^{2}}{2\sigma-\sigma^{2}+\delta^{2}}$$

where *δ* = |*p*_1_ – *p*_2_| and *σ* = *p*_1_ + *p*_2_. Without loss of generality, we assume that 1 ≤ *σ* ≤ 2. Three properties are of use: (*i*) MAF = 1 – *σ*/2; (*ii*) 0 ≤ 2*σ* –*σ*^2^ ≤ 1 for 1 ≤ *σ* ≤ 2; (*iii*) 0 ≤ *δ* ≤ (2 –*σ*)^2^. Rearranging Eq. (2), we deduce that

$$F=2\left[1-\frac{2\sigma-\sigma^{2}}{2\sigma-\sigma^{2}+\delta^{2}}\right]\leq2\left[1-\frac{\sigma(2-\sigma)}{\sigma(2-\sigma)+(2-\sigma)^{2}}\right]=2-\sigma$$

Since MAF = 1 – *σ*/2, the above inequality is equivalent to max(*F*) ≤ 2 MAF. In Supplementary Figure S1, we display the differences between the upper bounds of *F* derived here and that obtained in previous studies using Nei's *F* (Maruki *et al*, 2012; Jakobsson *et al*, 2013). It can be seen that *F* can only assume a very restrictive range of values when MAF is small.

The estimator of *F* proposed by Hudson et al. (1992; see also Keinan *et al*, 2007; Bhatia *et al*, 2013) was employed:

$$\widehat{F}=\frac{\widehat{\pi}_{B}-\widehat{\pi}_{S}}{\widehat{\pi}_{S}}$$

where $\widehat{\pi}_{B}$ and $\widehat{\pi}_{S}$ are estimates of *π_B_* and *π_S_* obtained from data. Eq. (4) can be calculated using information from a single SNP. To combine information from multiple SNPs, the following two methods were used (Weir and Cockerham, 1984; Bhatia *et al*, 2013):

$$F^{U}=\frac{1}{S}\sum_{i=1}^{S} \widehat{F}^{(i)}$$

and

$$F^{W}=\frac{\sum_{i=1}^{S} (\widehat{\pi}_{B}^{(i)}-\widehat{\pi}_{S}^{(i)})}{\sum_{i=1}^{S} \widehat{\pi}_{B}^{(i)}}$$

where *S* is the number of SNPs, and $\widehat{F}^{(i)}$, $\widehat{\pi}_{B}^{(i)}$ and $\widehat{\pi}_{S}^{(i)}$ are values of the terms defined in Eq. (4) obtained using data from the *i*-th SNP.

It should be noted that *F^U^* gives equal weight to all SNPs, whereas *F^W^* gives more weight to SNPs with higher expected levels of polymorphism. In other words, *F^U^* is expected to be more sensitive to the presence of SNPs with low MAFs, but *F^W^* is dominated by SNPs that are on average more polymorphic. To see this more explicitly, assume that we are combining information from two SNPs (i.e., *S* = 2). We add a subscript *j* to the symbols defined above to signify the locus under consideration, so that we have *p_jk_­*, *σ_j_*, and *δ_j_* = |*p_j_*_1_ – *p_j_*_2_|. We further assume that 2 > *σ*_1_ ≥ *σ*_2_ ≥ 1. Note that *σ_j_* are regarded as parameters (e.g., a SNP under strong selective constraints is expected to have a larger *σ* [i.e., a smaller MAF] than a neutral SNP). Some straightforward algebra leads to the following results: (*i*) max[*F*(*σ*_1_)] ≤ max[*F^U^*] ≤ max[*F*(*σ*_2_)]; (*ii*) max[*F*(*σ*_1_)] ≤ max[*F^W^*] ≤ max[*F*(*σ*_2_)], where max[*F*(*σ_j_*)] = 2 – *σ_j_* (these results hold when *S* > 2; proof not shown). To see the differential sensitivities to SNPs with small MAFs, we define Δ_1_(*U*) = max[*F^U^*] – max[*F*(*σ*_1_)], Δ_2_(*U*) = max[*F*(*σ*_2_)] – max[*F^U^*], Δ_1_(*W*) = max[*F^W^*] – max[*F*(*σ*_1_)], and Δ_2_(*W*) = max[*F*(*σ*_2_)] – max[*F^W^*]. Using Eq. (3), we show that Δ_1_(*U*)/Δ_2_(*U*) = 1, but Δ_1_(*W*)/Δ_2_(*W*) = (2 – *σ*_2_)/(2 – *σ*_1_) ≥ 1. Thus, the behaviour of *F^W^* is more akin to that of the more polymorphic SNP (i.e., max[*F^W^*] is closer to max[*F*(*σ*_2_)] than to max[*F*(*σ*_1_)]). As we will see later, this property of *F^W^* can lead to the masking of important signatures of evolution when SNPs with different properties are combined.

**Results**

*Genome-wide polymorphism patterns in crossover regions*

Table 1 presents summaries of polymorphism patterns for autosomal (A) and X-linked (X) loci situated in genomic regions where crossing-over occurs (crossover regions). For ease of presentation, we will refer to nucleotide diversity, *π*, calculated using 0-fold sites, 4-fold sites, and SI sites (positions 8-30 from the 5’ end of short introns ≤ 65bp) as *π*_0_, *π*_4_, and *π_SI_*, respectively; a similar notational convention will be used for other statistics. For both A and X, and in both the Rwandan (RG) and French (FR) samples, *π*_0_, Tajima's *D*_0_ (Tajima 1989), and MAF_0_ (minor allele frequency) are significantly smaller than the corresponding estimates obtained from 4-fold and SI sites (*P*_permutation_ < 0.001 in all cases), consistent with the well-known fact that most nonsynonymous mutations are deleterious (Pal *et al*, 2006; Eyre-Walker and Keightley, 2007), and are therefore kept at low frequencies in the population by purifying selection (Kimura, 1983). Previous studies have suggested that SI sites may be neutrally evolving (Halligan and Keightley, 2006; Parsch *et al*, 2010). In our data set, *π_SI_* seems to be somewhat smaller than *π*_4_, which may be due to the stringent data filtering procedure we employed (see Materials and Methods), or the higher GC content at 4-fold sites compared to intronic sites, which in turn is expected to result in an increased mutation rate in 4-fold sites (Singh *et al.*, 2005; Keightley *et al.*, 2009). There is, however, no statistically discernible difference with respect to either MAF or Tajima's *D* between 4-fold and SI sites (Table 1; *P*_permutation_ > 0.1 for both A and X).

The FR sample has a lower level of diversity than RG for all three types of sites (Table 1), reflecting a loss of genetic variation induced by population bottlenecks which are believed to have occurred as the species migrated out of Africa (Haddrill *et al.*, 2005b; Li and Stephan, 2006; Thornton and Andolfatto, 2006; Hutter *et al.*, 2007; Duchen *et al*, 2013). The difference in *π*_0_ between the two populations is somewhat smaller than those observed for *π*_4_ and *π_SI_* [e.g., on A, *π*_0_(FR)/*π*_0_(RG) = 0.83 *versus* *π*_4_(FR)/*π*_4_(RG) = 0.77]. This is probably because more 0-fold sites are under strong selective constraint, so that variants at these sites behave almost deterministically, and are therefore less sensitive to demographic changes (e.g., Zeng, 2013).

To inspect overall patterns of genetic differentiation between the RG and FR populations, we calculated *F_ST_* [abbreviated here as *F*; see Eq. (1) in Materials and Methods], as defined by Weir and Cockerham (1984), using the estimator of Hudson et al. (1992). Two approaches were employed to combine information over multiple SNPs: un-weighted mean *F* [Eq. (5)] and weighted mean *F* [Eq. (6)], which will be referred to as $F^{U}$ and $F^{W}$, respectively. Since most nonsynonymous mutations are likely to be deleterious, it is expected that levels of population differentiation at these selectively constrained sites should be lower than those at less constrained sites (e.g., 4-fold sites) (Barreiro *et al.*, 2008; Maruki *et al*, 2012). Surprisingly, values of $F_{0}^{W}$, estimated using either the autosomal or X-linked data, are not statistically different from those of either $F_{4}^{W}$ or $F_{SI}^{W}$ (Table 1; *P*_permutation_ > 0.1 in all cases). There is also no detectable difference between $F_{4}^{W}$ and $F_{SI}^{W}$ (*P*_permutation_ > 0.1 for both A and X). In contrast, $F_{0}^{U}$ was found to be significantly smaller than both $F_{4}^{U}$ and $F_{SI}^{U}$ (*P*_permutation_ < 0.001 for both A and X), while the differences between $F_{4}^{U}$ and $F_{SI}^{U}$ remain non-significant (*P*_permutation_ > 0.1 for both A and X). The patterns obtained from $F^{U}$ are therefore more compatible with the *a priori* expectation that 0-fold sites are on average more constrained than 4-fold and SI sites. We will investigate causes for the lack of difference between $F_{0}^{W}$ and either $F_{4}^{W}$ or $F_{SI}^{W}$ in a later section.

Several differences between A and X are of note (Table 1). Firstly, consistent with previous reports (Caracristi and Schlotterer, 2003; Hutter *et al*, 2007; Charlesworth, 2012b; Pool *et al*, 2012; Campos *et al.*, 2013), the X:A ratio in diversity at putatively neutral sites (i.e., 4-fold and SI sites) is about 1 in the RG population [*π*_4_(X)/*π*_4_(A) = 1.08 and *π_SI_*(X)/*π_SI_*(A) = 1.10], higher than the *null* expectation of 3/4. Secondly, the reduction in diversity in FR is more pronounced for X than A for all three types of sites [e.g., *π*_4_(FR)/*π*_4_(RG) = 0.41 and 0.77 for X and A, respectively], as reported in previous investigations (Caracristi and Schlotterer, 2003; Hutter *et al*, 2007). Finally, the extent of population differentiation at both 4-fold and SI sites, as measured by either $F^{U}$ or $F^{W}$, is significantly higher on the X than on A (*P*_permutation_ < 0.001 for all comparisons). This is probably largely driven by the greater reduction in diversity on the X in non-African populations, as values of *D_xy_*, the mean number of nucleotide substitutions between sequences taken from different subpopulations (Nei and Miller, 1990), are comparable between A and X in this study: *D_xy_*_,4_ = 1.65% and 1.64%, and *D_xy_*,*_SI_* = 1.51% and 1.58%. A systematic examination of possible causes of the apparent differences between A and X is beyond the scope of this study; the interested reader can refer to previous studies of this topic (Charlesworth, 2001; Pool and Nielsen, 2007; Singh *et al.*, 2007; Pool and Nielsen, 2008; Yukilevich *et al*, 2010; Charlesworth, 2012b; Campos *et al*, 2013). In what follows, results obtained from A and X will be presented separately.

*Limited evidence for selection on codon usage bias affecting patterns of population differentiation at 4-fold degenerate sites*

To investigate whether selection on codon usage bias (CUB) affects differentiation patterns at 4-fold sites, we first examined the relationship between $F_{4}^{U}$ and *Fop* (frequency of optimal codons), as the latter is well known to be correlated with the intensity of selection on CUB (reviewed in Hershberg and Petrov, 2008; Zeng and Charlesworth, 2009). Considering the large variance of the *F* estimators and the dearth of SNPs in individual genes, we grouped the genes into equal-sized bins with similar numbers of SNPs at 4-fold sites. As shown in Supplementary Figure S2A, *Fop* and $F_{4}^{U}$ are not correlated on A (Kendall's *τ* = -0.01, *P* > 0.1). On the X, some evidence for a weak negative correlation was obtained (Figure S2B), but it is not statistically significant (Kendall's *τ* = -0.6, *P* = 0.13). When $F_{4}^{W}$ was considered, no correlation was found on either A or X (Supplementary Figure S2E and S2F). To investigate this further, for the genes within each bin on the X, we tested whether $F_{4}^{U}$ differed from $F_{SI}^{U}$ statistically. Amongst the six bins, no evidence of a significant difference was found for the first four bins, whereas the differences were marginally significant for the last two bins with highest *Fop* (*P*_permutation_ = 0.04 and 0.05, respectively). Similarly, we did not detect any correlation between *K_S_* and either $F_{4}^{U}$ or $F_{4}^{W}$ (Supplementary Figure S2).

Overall, there is limited evidence that selection on CUB is strong enough to substantially alter patterns of genetic differentiation at 4-fold sites. Considering that 4-fold and SI sites in crossover regions are comparable with respect to both MAF and *F*, in what follows, we will use population differentiation patterns obtained from the two types of site as neutral standards, and will refer to them as putatively neutral sites.

*Evolutionarily conserved genes are under stronger purifying selection and have reduced F* *at 0-fold degenerate sites*

Genes in crossover regions were divided into equal-sized bins (with similar numbers of SNPs) based on their *K_A_* values between *D. melanogaster* and *D. yakuba*. We inspected polymorphism patterns in the RG sample as a function of *K_A_*; a qualitatively identical set of results were obtained using the FR sample (Supplementary Figure S3). On both A and X, *K_A_* was found to be significantly positively correlated with both *π*_0_ (Figures 1A and 1B; A: Kendall's *τ* = 0.989 and *P* < 0.001; X: Kendall's *τ* = 1 and *P* = 0.009) and Tajima's *D*_0_ (Figures 1C and 1D; A: Kendall's *τ* = 0.884, *P* < 0.001; X: Kendall's *τ* = 0.867 and *P* = 0.024). No statistically significant relationship was found when comparing *K_A_* with Tajima's *D*_4_ (Figures 1C and 1D; Kendall's *τ* = -0.2 and -0.333, *P* > 0.1, for X and A), although there is a negative correlation between *K_A_* and *π*_4_ on A (Figure 1A; Kendall's *τ* = -0.6, *P* < 0.001) (see also Andolfatto, 2007; Haddrill *et al.*, 2011). In particular, on both A and X, *π*_0_ and Tajima's *D*_0_ approach *π*_4_ and Tajima's *D*_4_, respectively, as *K_A_* increases. In contrast, values of *π*_4_ and Tajima's *D*_4_, regardless of the *K_A_* bin from which they were obtained, remain similar to the values of *π_SI_* and Tajima's *D_SI_*. These results suggest that 0-fold sites are under stronger constraints than 4-fold and SI sites, and that 0-fold sites in genes with smaller *K_A_* are, on average, under stronger purifying selection. We obtained the same results when we used the *D. simulans* genome as an outgroup (Supplementary Figure S4).

Figures 2A and 2B show that evolutionarily conserved genes have significantly smaller $F_{0}^{U}$ (A: Kendall's *τ* = 0.663, *P* < 0.001; X: Kendall's *τ* = 0.867, *P* = 0.02). Again, we obtained the same result when using *D. simulans* as the outgroup (Supplementary Figure S5). The pattern remains statistically significant for autosomes when $F_{0}^{W}$ was considered (Supplementary Figure S6). The reduction in *F*_0_ for genes with smaller *K_A_* is associated with a strong reduction in MAF_0_ (Figures 2C and 2D) and an increase in the proportion of 0-fold SNPs that are private to one of the two populations (Figures 2E and 2F), both of which are hallmarks of selection against deleterious mutations (cf., recent findings in humans; Nelson *et al.*, 2012; Fu *et al.*, 2013), and are expected to drive both *F^U^* and *F^W^* downwards, as shown in Materials and Methods (see also Maruki *et al*, 2012; Bhatia *et al*, 2013; Jakobsson *et al*, 2013). For the 4-fold sites on both A and X, no correlation with *K_A_* was observed for *F^U^*, *F^W^*, MAF, and the proportion of private SNPs (Figure 2; *P* > 0.1 in all cases based on Kendall's *τ*).

The data presented in Figures 1 and 2 suggests that the lack of difference between $F_{0}^{W}$ and either $F_{4}^{W}$ or $F_{SI}^{W}$ reported in the previous section is probably due to the fact that *F^W^* gives more weight to SNPs with higher expected levels of polymorphism (e.g., nearly neutral variants), as we have shown in Materials and Methods. In other words, when all 0-fold sites in crossover regions were analysed together (Table 1), the effects of purifying selection on a substantial fraction of 0-fold sites were probably masked by those 0-fold sites that are nearly neutrally evolving. Consequently, the overall distribution of $F_{0}^{W}$ appears non-distinguishable from those of $F_{4}^{W}$ and $F_{SI}^{W}$. In contrast, *F^U^* gives equal weight to all SNPs. Considering that the value of *F* when calculated using a single SNP is constrained by MAF [see Eq. (3) in Materials and Methods], *F^U^* is expected to be more sensitive to the action of purifying selection than *F^W^*, consistent with the observation reported above. In the Discussion, we will further explore the implications of these statistical properties of *F*, which arise when information from multiple SNPs is combined.

*Longer introns are under stronger selective constraints and are less differentiated*

In agreement with earlier findings (Haddrill *et al*, 2005a; Halligan and Keightley, 2006), longer introns tend to have lower divergence (*K*) between *D. melanogaster* and *D. simulans* (A: Kendall's *τ* = -0.635, *P* < 0.001; X: Kendall's *τ* = -0.486, *P* < 0.001; Figures 3A and 3B), probably as a result of the presence of functional elements that are subject to purifying selection (Bergman and Kreitman, 2001; Parsch, 2003; Andolfatto, 2005; Haddrill *et al*, 2005a; Halligan and Keightley, 2006; Casillas *et al*, 2007; Roy *et al*, 2010). Here, we report further support for this hypothesis by examining within-population polymorphism patterns as a function of intron length. Consistent with the action of purifying selection, longer introns have lower *π* (Figures 3C and 3D) and more negative Tajima's *D* (Figures 3E and 3F) compared to 4-fold and SI sites (similar results were observed in the FR sample; see Supplementary Figure S7). Interestingly, the patterns of divergence and polymorphism level off for introns longer than 2000 bp. Using the RG sample, the values of *π* and Tajima's *D* obtained from introns longer than 2000 bp are 0.0072 and -0.5476 for A, and 0.0076 and -0.9013 for X; all these values are substantially lower than the corresponding values observed at 4-fold and SI sites, but are higher than those obtained from 0-fold sites (see Table 1). Furthermore, the *K_A_* values for CDS in crossover regions between *D. melanogaster* and *D. simulans* are 0.015 and 0.018 for A and X, respectively, which are significantly smaller than the values of *K* for long introns > 2000 bp on A and X, which are 0.061 and 0.074, respectively (Mann-Whitney U test, *P* < 0.001). These results imply that long introns, especially those > 2000 bp, are more constrained than the 4-fold and SI sites, but probably contain fewer strongly selected sites than 0-fold sites.

Estimates of *F^W^*, when calculated using sites from introns more than 65 bp in length, were 0.171 and 0.283 for A and for X, respectively. None of these was found to be statistically different from the corresponding values estimating using 4-fold and SI sites reported in Table 1 (*P_permutation_* > 0.1 in all cases). *F^U^* for introns > 65 bp were 0.157 and 0.174 for A and X, respectively, both of which were significantly smaller than both $F_{SI}^{U}$ and $F_{4}^{U}$(*P_permutation_* < 0.001 in all cases). There is a clear negative relationship between *F^U^* and intron length (Figures 4A and 4B; for A and X, Kendall's *τ* = -0.356 and -0.364; *P* = 0.010 and *P* < 0.001, respectively), which mirrors that between MAF (or the prevalence of private SNPs) and intron length (Supplementary Figure S8), and is consistent with the expected effect of purifying selection on genetic differentiation between populations. The relationship between differentiation and intron length is weaker when *F^W^* was analysed (Supplementary Figure S8; for A and X, Kendall's *τ* = -0.271 and -0.146, and *P* = 0.05 and 0.16, respectively). These differences between *F^W^* and *F^U^* can be explained by the fact that fewer sites in introns > 65 bp are expected to be strongly selected compared to 0-fold sites. As discussed in the previous section, *F^W^*, which tends to reflect differentiation patterns at neutral sites in the data, is less likely to recover signatures of purifying selection compared to *F^U^*.

*Regions with reduced recombination tend to have higher F*

It is known that genomic regions that lack crossing over [non-crossover (NC) regions] have very different patterns of divergence and polymorphism than those seen in crossover (C) regions (Haddrill *et al.*, 2007; Betancourt *et al.*, 2009; Arguello *et al.*, 2010; Campos *et al*, 2012; Campos *et al.*, 2014). In Table 2, we present summary statistics of the NC data pertinent to the current study (see Materials and Methods for a list of the NC regions considered). It can be seen that, for both 0-fold and 4-fold sites, values of *F* in NC regions are generally higher than those obtained using the same type of site in C regions, regardless of the way in which information from multiple SNPs was combined. Specifically, the average *K_A_* to *D. yakuba* is about 0.05 for the NC loci (Campos *et al*, 2012). $F_{0}^{U}$ calculated using autosomal and X-linked NC data are 0.1817 and 0.3012, respectively (Table 2), higher than the values of 0.1569 and 0.1685 for autosomal and X-linked genes in C regions spanning the same *K_A_* values (Figures 2A and 2B; *P_permutation_* = 0.05 for A and *P_permutation_* < 0.001 for X).

It should be noted that the elevation in *F* in NC regions is probably caused by an extreme reduction in within-population diversity induced by tight linkage between a large number of selected sites (Table 2; Kaiser and Charlesworth, 2009; O'Fallon *et al.*, 2010; Seger *et al.*, 2010; Zeng and Charlesworth, 2010). This is because *F* is a relative measure of differentiation [see Eq. (1)], and therefore all else being equal, *F* is expected to be elevated by forces that reduce within-population diversity [i.e., *π_S_* in Eq. (1)], irrespective of whether diversifying selection or reduced gene flow has affected the genomic region under study (Charlesworth, 1998; Noor and Bennett, 2009).

To further examine the effects of selection at linked sites, we inspect the correlation between *F* at putatively neutral sites and local recombination rates in C regions. Figure 5 presents results based on autosomal loci, where it can be seen that $F_{4}^{U}$ is reduced with more frequent recombination (Kendall's *τ* = -0.474, *P* = 0.004; the data point obtained from the NC regions was not included in the calculation). However, there is no statistically significant relationship between recombination rate and $F_{SI}^{U}$ (Figure 5B; Kendall's *τ* = -0.179 and *P* = 0.28). Weak negative correlations were also found on the X chromosome for 4-fold and SI sites (Supplementary Figure S9). The patterns remained unchanged when *F^W^* was used (Supplementary Figure S10).

**Discussion**

By using the high-quality data provided by the *Drosophila* Population Genomics Project, we have found that evolutionary conserved regions (i.e., genes with lower *K_A_* and longer introns) show clear evidence of more intense on-going purifying selection than less conserved genomic regions, which can be detected by analysing patterns of genetic variation both within and between subpopulations. The negative correlation between *π* and intron length reported in Figure 3 extends the study by Parsch et al. (2010) who examined a much smaller data set and did not find evidence of such a correlation. Since we did not find support for a correlation between local recombination rate and intron length (Kendall's *τ* = -0.004 and 0.011 for A and X, respectively, and *P* > 0.1 in both cases; Supplementary Figure S11) (c.f., Carvalho and Clark, 1999; Comeron and Kreitman, 2000), the relationship is unlikely to be driven by the well-known positive correlation between diversity and recombination. It is unclear why the effect of intron length levels off for introns longer than 2000bp. Analysis of theoretical models (e.g., Ometto et al. 2005) and improved annotation of non-coding functional elements (e.g., Roy et al. 2010) are both needed to solve this problem. Finally, there is evidence that the severe reduction in within-population diversity in non-crossover regions of the genome induced by tight linkage between selected sites has led to elevated *F_ST_* values, but there is limited support for this effect in crossover regions.

*Purifying selection as a major determinant of population differentiation*

Our analysis reveals (*i*) a positive correlation between *K_A_* and *F*_0_ (Figure 2) and (*ii*) a negative correlation between intron length and *F* calculated using intronic sites (Figure 4). After examining other aspects of polymorphism and differentiation patterns (Figures 1 and 3), we suggest that the observations can be most readily explained by differential intensity of purifying selection acting on different parts of the genome. Similar observations have also been reported in humans (Barreiro *et al*, 2008; Maruki *et al*, 2012), suggesting the universal importance of purifying selection as a factor that shapes genetic differentiation between populations.

It should be noted that the above conclusion is not inconsistent with the existence of outlier loci with unusually high *F*, which may have been caused by diversifying selection (Turner *et al*, 2008; Yukilevich *et al*, 2010; Kolaczkowski *et al*, 2011; Fabian *et al*, 2012; Langley *et al*, 2012; Pool *et al*, 2012; Campo *et al*, 2013). Our analysis intends to detect forces with large-scale effects (there are typically hundreds of genes in each of the bins in our analysis), and is therefore unlikely to respond to processes that have more localised effects in the genome. In fact, it has been suggested that the number of loci contributing to differences between populations may be relatively small (Yukilevich *et al*, 2010; Fabian *et al*, 2012). For example, after taking into account the confounding effects of complex demography and correcting for multiple testing, only four loci had strong statistical support for being driven to high levels of differentiation by diversifying selection between North American and African populations of *D. melanogaster* (Yukilevich *et al*, 2010). Furthermore, in line with the low level of linkage disequilibrium (LD) in the *D. melanogaster* genome (e.g., Pool *et al*, 2012), previous genome scan studies have shown that most candidate variants that show evidence of involving in local adaptation only affect differentiation patterns in its immediate neighbourhood, typically on the order of the size of a gene (Kolaczkowski *et al*, 2011; Fabian *et al*, 2012). Finally, we have focused on protein-coding regions and introns whereas a substantial number of previously-found candidate loci fall within intergenic regions.

A noticeable exception is chromosome 3R, in which the cosmopolitan inversion *In(3R)P* is situated. Multiple studies concerning differentiation between various *D. melanogaster* populations have found that chromosome 3R has a disproportionally large number of candidate loci, especially within the *In(3R)P* region, and that these candidate variants tend to affect differentiation patterns in a larger genomic neighbourhood (Kolaczkowski *et al*, 2011; Fabian *et al*, 2012). To further test the robustness of our results, we repeated the analysis leading to Figure 2A by removing all genes on chromosome 3R, and found that the pattern remains unchanged (Supplementary Figure S12). In summary, it is unlikely that highly differentiated regions driven by adaptive changes have made a substantial contribution to our observations.

*The relationship between F and recombination*

As pointed out previously (Charlesworth, 1998; Noor and Bennett, 2009), forces that reduce within-population diversity can lead to elevated *F_ST_* values in the absence of diversifying selection and restricted gene flow. Hence, in light of the lack of evidence of adaptive evolution in non-crossover regions of the *D. melanogaster* genome (Betancourt *et al*, 2009; Arguello *et al*, 2010; Campos *et al*, 2014), the high *F* values obtained from NC regions is probably a result of the diversity-reducing effect of linkage between selected sites, which is often referred to Hill-Robertson interference or HRI (Hill and Robertson, 1966; Comeron *et al.*, 2008; Sella *et al.*, 2009; Charlesworth, 2012a; Cutter and Payseur, 2013). Within the C regions, although negative correlations between *F* at putatively neutral sites and local recombination rate, as predicted by the HRI theory, were observed (Figures 5, S9 and S10), these patterns are weak and often non-significant. Langley et al. (2012) also reported weak negative correlations between a different measure of genetic differentiation and fine-scale recombination rates estimated from LD patterns, but the relationship was inconsistent between chromosome arms and was sometimes weakly positive when broad-scale recombination rates were used.

The weak association between *F* and recombination in C regions is somewhat surprising given that both *π*_4_ and *π_SI_* are clearly positively correlated with local recombination rates in both the RG and FR populations (Supplementary Figure S13). A possible explanation is that, since hitchhiking effects induced by both positive and negative selection can lead to an excess of low-frequency variants at linked neutral sites (Charlesworth *et al.*, 1993; Braverman *et al.*, 1995; Zeng and Charlesworth, 2011), the negative correlation between *F* and recombination may be weakened, if rare variants are more common in low-recombination regions, as these variants tend to lower *F* [see Eq. (3)]. Tajima's *D* is somewhat more negative in autosomal C regions with reduced recombination (Supplementary Figure S14), but it is hard to determine to what extent this has contributed to the observations in Figures 5 and S9, especially when noting that NC regions have more negative Tajima's *D* and yet higher *F_ST_* values. Further research that takes into account HRI, demography, and statistical properties of estimators of *F* (see below) is needed to clarify the matter.

*The importance of sampling strategy regarding using F_ST_ to study population differentiation*

As is the case for other definitions of *F_ST_* (Maruki *et al*, 2012; Jakobsson *et al*, 2013), Weir and Cockerham's *F_ST_* can only take a very restricted range of values when MAF is small [max(*F_ST_*) ≤ 2 MAF; Supplementary Figure S1]. When information is combined across SNPs, the weighted mean *F_ST_* (*F^W^*) is likely to be dominated by SNPs that are more polymorphic (i.e., those having a higher expected MAF). This can lead to the masking of signals of purifying selection, as we have shown above. Thus, *F^W^* may be a better choice when the intention is to ascertain the overall level of genetic differentiation. In this case, as long as the data contains a substantial number of putatively neutrally evolving variants, a reasonably accurate estimate can be obtained, even in the presence of sites under strong selective constraints. In contrast, the unweighted mean *F_ST_* (*F^U^*) gives equal weight to all SNPs, and is more responsive to the presence of rare variants (e.g., those under purifying selection). These considerations, as well as the recommendations proposed by Bhatia et al. (2013), suggest that care should be exercised when deciding which sampling strategy is most appropriate for the question in hand.

**Acknowledgements**

We are grateful to the *Drosophila* Population Genomics Project (DPGP) for making these data available. We also thank Brian Charlesworth and three anonymous reviewers for useful discussions and comments. BCJ would like to thank Quentin Geissmann for time spent helping him to become a better programmer. This work was supported by a PhD studentship to BCJ, jointly funded by the National Environmental Research Council (NERC) (NE/H524881/1 and NE/K500914/1) and the Department of Animal and Plant Sciences, University of Sheffield; and a Biotechnology and Biological Sciences Research Council (BBSRC) grant (BB/K000209/1) awarded to KZ. This work has made use of High Performance Computing Cluster, Iceberg, at the University of Sheffield.

**Conflict of Interest**

The authors declare no conflict of interest.

**Data Archiving**

All genetic data analysed in this study are publically available.

**References**

Andolfatto P (2005). Adaptive evolution of non-coding DNA in *Drosophila*. *Nature* **437:** 1149-1152.

Andolfatto P (2007). Hitchhiking effects of recurrent beneficial amino acid substitutions in the *Drosophila* *melanogaster* genome. *Genome Res* **17:** 1755-1762.

Arguello JR, Zhang Y, Kado T, Fan C, Zhao R, Innan H *et al* (2010). Recombination yet inefficient selection along the *Drosophila melanogaster* subgroup's fourth chromosome. *Mol Biol Evol* **27:** 848-861.

Barreiro LB, Laval G, Quach H, Patin E, Quintana-Murci L (2008). Natural selection has driven population differentiation in modern humans. *Nature Genetics* **40:** 340-345.

Beaumont MA (2005). Adaptation and speciation: what can F_ST_ tell us? *Trends Ecol Evol* **20:** 435-440.

Beaumont MA, Balding DJ (2004). Identifying adaptive genetic divergence among populations from genome scans. *Mol Ecol* **13:** 969-980.

Beaumont MA, Nichols RA (1996). Evaluating loci for use in the genetic analysis of population structure. *P Roy Soc B-Biol Sci* **263:** 1619-1626.

Bergman CM, Kreitman M (2001). Analysis of conserved noncoding DNA in *Drosophila* reveals similar constraints in intergenic and intronic sequences. *Genome Res* **11:** 1335-1345.

Betancourt AJ, Welch JJ, Charlesworth B (2009). Reduced effectiveness of selection caused by a lack of recombination. *Curr Biol* **19:** 655-660.

Bhatia G, Patterson N, Sankararaman S, Price AL (2013). Estimating and interpreting F_ST_: the impact of rare variants. *Genome Res* **23:** 1514-1521.

Braverman JM, Hudson RR, Kaplan NL, Langley CH, Stephan W (1995). The hitchhiking effect on the site frequency spectrum of DNA polymorphisms. *Genetics* **140:** 783-796.

Campo D, Lehmann K, Fjeldsted C, Souaiaia T, Kao J, Nuzhdin SV (2013). Whole-genome sequencing of two North American *Drosophila melanogaster* populations reveals genetic differentiation and positive selection. *Mol Ecol* **22:** 5084-5097.

Campos JL, Charlesworth B, Haddrill PR (2012). Molecular evolution in nonrecombining regions of the *Drosophila melanogaster* genome. *Genome Biol Evol* **4:** 278-288.

Campos JL, Halligan DL, Haddrill PR, Charlesworth B (2014). The relation between recombination rate and patterns of molecular evolution and variation in *Drosophila melanogaster*. *Mol Biol Evol*.

Campos JL, Zeng K, Parker DJ, Charlesworth B, Haddrill PR (2013). Codon usage bias and effective population sizes on the X chromosome versus the autosomes in *Drosophila melanogaster*. *Mol Biol Evol* **30:** 811-823.

Caracristi G, Schlotterer C (2003). Genetic differentiation between American and European *Drosophila melanogaster* populations could be attributed to admixture of African alleles. *Mol Biol Evol* **20:** 792-799.

Carvalho AB, Clark AG (1999). Intron size and natural selection. *Nature* **401:** 344.

Casillas S, Barbadilla A, Bergman CM (2007). Purifying selection maintains highly conserved noncoding sequences in *Drosophila*. *Mol Biol Evol* **24:** 2222-2234.

Charif D, Lobry J (2007). SeqinR 1.0-2: A contributed package to the R Project for Statistical Computing devoted to biological sequences retrieval and analysis. In: Bastolla U, Porto M, Roman HE and Vendruscolo M (eds) *Structural Approaches to Sequence Evolution*. Springer Berlin Heidelberg, pp 207-232.

Charlesworth B (1998). Measures of divergence between populations and the effect of forces that reduce variability. *Mol Biol Evol* **15:** 538-543.

Charlesworth B (2001). The effect of life-history and mode of inheritance on neutral genetic variability. *Genet Res* **77:** 153-166.

Charlesworth B (2012a). The effects of deleterious mutations on evolution at linked sites. *Genetics* **190:** 5-22.

Charlesworth B (2012b). The role of background selection in shaping patterns of molecular evolution and variation: evidence from variability on the *Drosophila* X chromosome. *Genetics* **191:** 233-246.

Charlesworth B, Charlesworth D (2010). *Elements of evolutionary genetics*. Roberts & Company Publishers: Greenwood Village.

Charlesworth B, Charlesworth D, Barton NH (2003). The effects of genetic and geographic structure on neutral variation. *Annu Rev Ecol Evol S* **34:** 99-125.

Charlesworth B, Morgan MT, Charlesworth D (1993). The effect of deleterious mutations on neutral molecular variation. *Genetics* **134:** 1289-1303.

Comeron JM, Kreitman M (2000). The correlation between intron length and recombination in *Drosophila*. Dynamic equilibrium between mutational and selective forces. *Genetics* **156:** 1175-1190.

Comeron JM, Ratnappan R, Bailin S (2012). The many landscapes of recombination in *Drosophila melanogaster*. *PLoS Genet* **8:** e1002905.

Comeron JM, Williford A, Kliman RM (2008). The Hill-Robertson effect: evolutionary consequences of weak selection and linkage in finite populations. *Heredity* **100:** 19-31.

Cutter AD, Choi JY (2010). Natural selection shapes nucleotide polymorphism across the genome of the nematode Caenorhabditis briggsae. Genome Res **20:** 1103-1111

Cutter AD, Payseur BA (2013). Genomic signatures of selection at linked sites: unifying the disparity among species. *Nat Rev Genet* **14:** 262-274.

David JR, Capy P (1988). Genetic variation of *Drosophila melanogaster* natural populations. *Trends Genet* **4:** 106-111.

Duchen P, Zivkovic D, Hutter S, Stephan W, Laurent S (2013). Demographic inference reveals African and European admixture in the North American *Drosophila melanogaster* population. *Genetics* **193:** 291-301.

Excoffier L, Hofer T, Foll M (2009). Detecting loci under selection in a hierarchically structured population. *Heredity* **103:** 285-298.

Excoffier L, Smouse PE, Quattro JM (1992). Analysis of molecular variance inferred from metric distances among DNA haplotypes: application to human mitochondrial DNA restriction data. *Genetics* **131:** 479-491.

Eyre-Walker A, Keightley PD (2007). The distribution of fitness effects of new mutations. *Nat Rev Genet* **8:** 610-618.

Eyre-Walker A, Keightley PD (2009). Estimating the rate of adaptive molecular evolution in the presence of slightly deleterious mutations and population size change. *Mol Biol Evol* **26:** 2097-2108.

Fabian DK, Kapun M, Nolte V, Kofler R, Schmidt PS, Schlotterer C *et al* (2012). Genome-wide patterns of latitudinal differentiation among populations of *Drosophila melanogaster* from North America. *Mol Ecol* **21:** 4748-4769.

Fiston-Lavier AS, Singh ND, Lipatov M, Petrov DA (2010). *Drosophila melanogaster* recombination rate calculator. *Gene* **463:** 18-20.

Foll M, Gaggiotti O (2008). A genome-scan method to identify selected loci appropriate for both dominant and codominant markers: a Bayesian perspective. *Genetics* **180:** 977-993.

Fu W, O'Connor TD, Jun G, Kang HM, Abecasis G, Leal SM *et al* (2013). Analysis of 6,515 exomes reveals the recent origin of most human protein-coding variants. *Nature* **493:** 216-220.

Haddrill PR, Charlesworth B, Halligan DL, Andolfatto P (2005a). Patterns of intron sequence evolution in *Drosophila* are dependent upon length and GC content. *Genome Biol* **6:** R67.

Haddrill PR, Halligan DL, Tomaras D, Charlesworth B (2007). Reduced efficacy of selection in regions of the *Drosophila* genome that lack crossing over. *Genome Biol* **8:** R18.

Haddrill PR, Thornton KR, Charlesworth B, Andolfatto P (2005b). Multilocus patterns of nucleotide variability and the demographic and selection history of *Drosophila melanogaster* populations. *Genome Res* **15:** 790-799.

Haddrill PR, Zeng K, Charlesworth B (2011). Determinants of synonymous and nonsynonymous variability in three species of *Drosophila*. *Mol Biol Evol* **28:** 1731-1743.

Halligan DL, Keightley PD (2006). Ubiquitous selective constraints in the *Drosophila* genome revealed by a genome-wide interspecies comparison. *Genome Res* **16:** 875-884.

Hershberg R, Petrov DA (2008). Selection on codon bias. *Annu Rev Genet* **42:** 287-299.

Hey J, Machado CA (2003). The study of structured populations—new hope for a difficult and divided science. *Nat Rev Genet* **4:** 535-543.

Hill WG, Robertson A (1966). The effect of linkage on limits to artificial selection. *Genet Res* **8:** 269-294.

Holsinger KE, Weir BS (2009). Genetics in geographically structured populations: defining, estimating and interpreting F_ST_. *Nat Rev Genet* **10:** 639-650.

Hu TT, Eisen MB, Thornton KR, Andolfatto P (2013). A second-generation assembly of the *Drosophila simulans* genome provides new insights into patterns of lineage-specific divergence. *Genome Res* **23:** 89-98.

Hudson RR, Slatkin M, Maddison WP (1992). Estimation of levels of gene flow from DNA sequence data. *Genetics* **132:** 583-589.

Hutter S, Li H, Beisswanger S, De Lorenzo D, Stephan W (2007). Distinctly different sex ratios in African and European populations of *Drosophila melanogaster* inferred from chromosomewide single nucleotide polymorphism data. *Genetics* **177:** 469-480.

Jakobsson M, Edge MD, Rosenberg NA (2013). The relationship between F_ST_ and the frequency of the most frequent allele. *Genetics* **193:** 515-528.

Kaiser VB, Charlesworth B (2009). The effects of deleterious mutations on evolution in non-recombining genomes. *Trends Genet* **25:** 9-12.

Katoh K, Misawa K, Kuma K, Miyata T (2002). MAFFT: a novel method for rapid multiple sequence alignment based on fast Fourier transform. *Nucleic Acids Res* **30:** 3059-3066.

Keightley PD, Eyre-Walker A (2007). Joint inference of the distribution of fitness effects of deleterious mutations and population demography based on nucleotide polymorphism frequencies. *Genetics* **177:** 2251-2261.

Keightley PD, Trivedi U, Thomson M, Oliver F, Kumar S, Blaxter ML (2009). Analysis of the genome sequences of three *Drosophila melanogaster* spontaneous mutation accumulation lines. *Genome Res* **19:** 1195-1201.

Keinan A, Mullikin JC, Patterson N, Reich D (2007). Measurement of the human allele frequency spectrum demonstrates greater genetic drift in East Asians than in Europeans. *Nat Genet* **39:** 1251-1255.

Keinan A, Mullikin JC, Patterson N, Reich D (2009). Accelerated genetic drift on chromosome X during the human dispersal out of Africa. *Nat Genet* **41:** 66-70.

Kimura M (1980). A simple method for estimating evolutionary rates of base substitutions through comparative studies of nucleotide sequences. *J Mol Evol* **16:** 111-120.

Kimura M (1983). *The Neutral Theory of Molecular Evolution*. Cambridge University Press: Cambridge.

Kolaczkowski B, Kern AD, Holloway AK, Begun DJ (2011). Genomic differentiation between temperate and tropical Australian populations of *Drosophila melanogaster*. *Genetics* **187:** 245-260.

Langley CH, Stevens K, Cardeno C, Lee YC, Schrider DR, Pool JE *et al* (2012). Genomic variation in natural populations of *Drosophila melanogaster*. *Genetics* **192:** 533-598.

Li H, Stephan W (2006). Inferring the demographic history and rate of adaptive substitution in *Drosophila*. *PLoS Genet* **2:** e166.

Li WH (1993). Unbiased Estimation of the Rates of Synonymous and Nonsynonymous Substitution. *J Mol Evol* **36:** 96-99.

Maruki T, Kumar S, Kim Y (2012). Purifying selection modulates the estimates of population differentiation and confounds genome-wide comparisons across single-nucleotide polymorphisms. *Mol Biol Evol* **29:** 3617-3623.

Nagylaki T (1998). Fixation indices in subdivided populations. *Genetics* **148:** 1325-1332.

Nei M (1973). Analysis of gene diversity in subdivided populations. *P Natl Acad Sci USA* **70:** 3321-3323.

Nei M, Miller JC (1990). A simple method for estimating average number of nucleotide substitutions within and between populations from restriction data. *Genetics* **125:** 873-879.

Nelson MR, Wegmann D, Ehm MG, Kessner D, St Jean P, Verzilli C *et al* (2012). An abundance of rare functional variants in 202 drug target genes sequenced in 14,002 people. *Science* **337:** 100-104.

Noor MA, Bennett SM (2009). Islands of speciation or mirages in the desert? Examining the role of restricted recombination in maintaining species. *Heredity* **103:** 439-444.

O'Fallon BD, Seger J, Adler FR (2010). A continuous-state coalescent and the impact of weak selection on the structure of gene genealogies. *Mol Biol Evol* **27:** 1162-1172.

Ometto L, Stephan W, De Lorenzo, D (2005). Insertion/deletion and nucleotide polymorphism data reveal contraints in *Drosophila melanogaster* introns and intergenic regions. *Genetics* **169:** 1521-1527.

Pal C, Papp B, Lercher MJ (2006). An integrated view of protein evolution. *Nat Rev Genet* **7:** 337-348.

Paradis E (2010). pegas: an R package for population genetics with an integrated-modular approach. *Bioinformatics* **26:** 419-420.

Paradis E, Claude J, Strimmer K (2004). APE: Analyses of Phylogenetics and Evolution in R language. *Bioinformatics* **20:** 289-290.

Parsch J (2003). Selective constraints on intron evolution in *Drosophila*. *Genetics* **165:** 1843-1851.

Parsch J, Novozhilov S, Saminadin-Peter SS, Wong KM, Andolfatto P (2010). On the utility of short intron sequences as a reference for the detection of positive and negative selection in *Drosophila*. *Mol Biol Evol* **27:** 1226-1234.

Peden JF (1999). Analysis of codon usage. PhD thesis, University of Nottingham, UK. Available from: http://codonw.source-forge.net/

Pool JE, Corbett-Detig RB, Sugino RP, Stevens KA, Cardeno CM, Crepeau MW *et al* (2012). Population Genomics of sub-saharan *Drosophila melanogaster*: African diversity and non-African admixture. *PLoS Genet* **8:** e1003080.

Pool JE, Nielsen R (2007). Population size changes reshape genomic patterns of diversity. *Evolution* **61:** 3001-3006.

Pool JE, Nielsen R (2008). The impact of founder events on chromosomal variability in multiply mating species. *Mol Biol Evol* **25:** 1728-1736.

Roy S, Ernst J, Kharchenko PV, Kheradpour P, Negre N, Eaton ML *et al* (2010). Identification of Functional Elements and Regulatory Circuits by *Drosophila* modENCODE. *Science* **330:** 1787-1797.

Schaeffer SW (2002). Molecular population genetics of sequence length diversity in the *Adh* region of *Drosophila pseudoobscura*. *Genet Res* **80:** 163-175.

Schneider A, Charlesworth B, Eyre-Walker A, Keightley PD (2011). A method for inferring the rate of occurrence and fitness effects of advantageous mutations. *Genetics* **189:** 1427-1437.

Seger J, Smith WA, Perry JJ, Hunn J, Kaliszewska ZA, Sala LL *et al* (2010). Gene genealogies strongly distorted by weakly interfering mutations in constant environments. *Genetics* **184:** 529-545.

Sella G, Petrov DA, Przeworski M, Andolfatto P (2009). Pervasive natural selection in the *Drosophila* genome? *PLoS Genet* **5:** e1000495.

Singh ND, Arndt PF, Petrov DA (2005). Genomic heterogeneity of background substitutional patterns in *Drosophila melanogaster*. *Genetics* **169:** 709-722.

Singh ND, Macpherson JM, Jensen JD, Petrov DA (2007). Similar levels of X-linked and autosomal nucleotide variation in African and non-African populations of *Drosophila melanogaster*. *BMC Evol Biol* **7:** 202.

Slatkin M (1995). A measure of population subdivision based on microsatellite allele frequencies. *Genetics* **139:** 457-462.

Stephan W, Li H (2007). The recent demographic and adaptive history of *Drosophila melanogaster*. *Heredity* **98:** 65-68.

Tajima F (1989). Statistical method for testing the neutral mutation hypothesis by DNA polymorphism. *Genetics* **123:** 585-595.

Thornton K, Andolfatto P (2006). Approximate Bayesian inference reveals evidence for a recent, severe bottleneck in a Netherlands population of *Drosophila melanogaster*. *Genetics* **172:** 1607-1619.

Turner TL, Levine MT, Eckert ML, Begun DJ (2008). Genomic analysis of adaptive differentiation in *Drosophila melanogaster*. *Genetics* **179:** 455-473.

Weir BS, Cockerham CC (1984). Estimating F-Statistics for the Analysis of Population-Structure. *Evolution* **38:** 1358-1370.

Weir BS, Hill WG (2002). Estimating F-statistics. *Annu Rev Genet* **36:** 721-750.

Wright S (1951). The Genetical Structure of Populations. *Ann Eugenic* **15:** 323-354.

Wu CI (2001). The genic view of the process of speciation. *J Evolution Biol* **14:** 851-865.

Yukilevich R, Turner TL, Aoki F, Nuzhdin SV, True JR (2010). Patterns and processes of genome-wide divergence between North American and African *Drosophila melanogaster*. *Genetics* **186:** 219-239.

Zeng K (2013). A coalescent model of background selection with recombination, demography and variation in selection coefficients. *Heredity* **110:** 363-371.

Zeng K, Charlesworth B (2009). Estimating selection intensity on synonymous codon usage in a nonequilibrium population. *Genetics* **183:** 651-662.

Zeng K, Charlesworth B (2010). The effects of demography and linkage on the estimation of selection and mutation parameters. *Genetics* **186:** 1411-1424.

Zeng K, Charlesworth B (2011). The joint effects of background selection and genetic recombination on local gene genealogies. *Genetics* **189:** 251-266.

**Table 1.** Summary statistics for loci in crossover (C) regions.

|  |  |  | Within population ^a^ | | |  | Between populations ^b^ | | |
| --- | --- | --- | --- | --- | --- | --- | --- | --- | --- |
| Chr | Site |  | Pop. ^c^ | *π* | Tajima's *D* |  | MAF | *F^U^* | *F^W^* |
| A | 0-fold |  | RG | 0.0012 | -0.8397 |  | 0.1222 | 0.1516 | 0.1709 |
|  |  |  | FR | 0.0010 | -0.2586 |  |  |  |  |
|  | 4-fold |  | RG | 0.0154 | -0.1069 |  | 0.1653 | 0.1684 | 0.1743 |
|  |  |  | FR | 0.0119 | 0.1116 |  |  |  |  |
|  | SI ^d^ |  | RG | 0.0145 | -0.1380 |  | 0.1630 | 0.1677 | 0.1766 |
|  |  |  | FR | 0.0113 | 0.1413 |  |  |  |  |
| X | 0-fold |  | RG | 0.0012 | -1.1907 |  | 0.1073 | 0.1653 | 0.2924 |
|  |  |  | FR | 0.0005 | -0.2293 |  |  |  |  |
|  | 4-fold |  | RG | 0.0166 | -0.4679 |  | 0.1367 | 0.1903 | 0.2879 |
|  |  |  | FR | 0.0068 | 0.1412 |  |  |  |  |
|  | SI ^d^ |  | RG | 0.0160 | -0.4561 |  | 0.1379 | 0.2033 | 0.3173 |
|  |  |  | FR | 0.0061 | 0.3414 |  |  |  |  |

^a^ Summary statistics calculated using data from within a subpopulation for the type of site under consideration.

^b^ Summary statistics calculated using data from both subpopulations for the type of site under consideration. The *F*-statistics are defined by Eqs. (5) and (6).

^c^ Population of origin; RG, Rwandan; FR, French.

^d^ Sites from 8-30bp regions of short introns ≤ 65bp.

**Table 2.** Summary statistics for loci in non-crossover (NC) regions.

|  |  |  | Within population | | |  | Between populations | | |
| --- | --- | --- | --- | --- | --- | --- | --- | --- | --- |
| Chr | Site |  | Pop. | *π* | Tajima's *D* |  | MAF | *F^U^* | *F^W^* |
| A | 0-fold |  | RG | 0.00036 | -0.6737 |  | 0.1152 | 0.1817 | 0.2302 |
|  |  |  | FR | 0.00032 | -0.7098 |  |  |  |  |
|  | 4-fold |  | RG | 0.00129 | -0.5274 |  | 0.1208 | 0.1906 | 0.2281 |
|  |  |  | FR | 0.00122 | -0.5417 |  |  |  |  |
| X | 0-fold |  | RG | 0.00056 | -0.6392 |  | 0.1556 | 0.3012 | 0.5673 |
|  |  |  | FR | 0.00023 | -0.3126 |  |  |  |  |
|  | 4-fold |  | RG | 0.00327 | -0.0084 |  | 0.1395 | 0.2323 | 0.3485 |
|  |  |  | FR | 0.00090 | 0.2069 |  |  |  |  |

The statistics were obtained in the same way as in Table 1; see Materials and Methods for more details.

**Titles and legends to figures**

**Figure 1. Polymorphism patterns within 17 Rwandan *D. melanogaster* lines for coding sequence (CDS) binned by *K_A_*value (to *D. yakuba*), and for sites in the 8-30 bp regions of short introns ≤ 65 bp (SI sites).** (A) Nucleotide diversity (*π*) for autosomal CDS where crossing-over occurs (CDS-C) and (B) X-linked CDS-C regions; (C) Tajima’s *D* for autosomal CDS-C regions and (D) X-linked CDS-C regions. The x-axes show the maximum *K_A_* value in each bin. Symbols: 0-fold degenerate sites—open circles; 4-fold degenerate sites—open triangles; SI sites—open red squares.

**Figure 2. Differentiation patterns between 7 French and 17 Rwandan *D. melanogaster* lines for coding sequence (CDS) binned by *K_A_* value (to *D. yakuba*), and for SI sites.** (A) Unweighted mean *F_ST_* [*F^U^*; Eq. (5)] for autosomal coding CDS where crossing-over occurs (CDS-C) and (B) X-linked CDS-C regions; (C) population-average minor allele frequency (MAF) for autosomal CDS-C regions and (D) X-linked CDS-C regions; (E) the proportion of SNPs per bin in which one allele was private to one of the *D. melanogaster* populations for autosomal CDS-C regions and (F) X-linked CDS-C regions. Symbols: 0-fold degenerate sites—open circles; 4-fold degenerate sites—open triangles; SI sites—open red squares.

**Figure 3. Divergence and polymorphism patterns for intronic sites binned by intron length.** (A) Divergence (*K*) between *D. melanogaster* and *D. simulans* for autosomal introns and (B) X-linked introns; (C) nucleotide diversity (*π*) for autosomal introns and (D) X-linked introns; (E) Tajima’s *D* for autosomal introns and (F) X-linked introns. The x-axes display the maximum intron length in each bin. Note that the number of SNPs in each autosomal intron bin is roughly the same as that in the autosomal SI bin; the same applies to the X-linked data. Symbols: Long intronic sites—open circles; positions 8-30bp sites of short introns ≤ 65 bp (SI sites)—open red squares.

**Figure 4. Differentiation between 7 French and 17 Rwandan *D. melanogaster* lines for long intronic sites binned by intron length.** (A) Unweighted mean *F_ST_* [*F^U^*; Eq. (5)] for autosomal introns and (B) X-linked introns. Symbols: Long intronic sites—open circles; SI sites—open red squares.

**Figure 5. Differentiation between 7 French and 17 Rwandan *D. melanogaster* lines for 4-fold degenerate sites and SI sites in crossover regions as a function of local recombination rate.** (A) *F^U^* for autosomal CDS regions and (B) autosomal SI regions.

**Figure 1**.

| 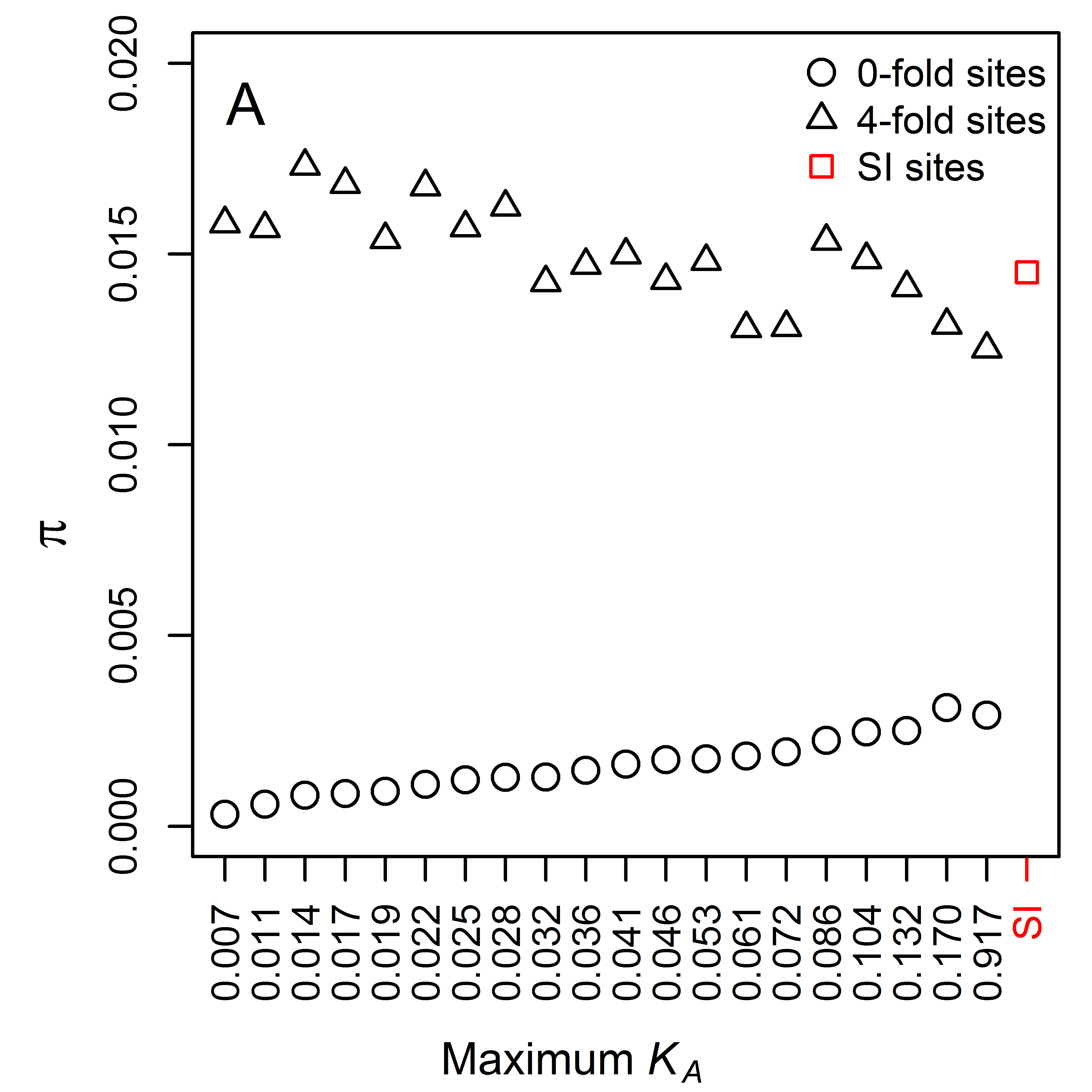 | 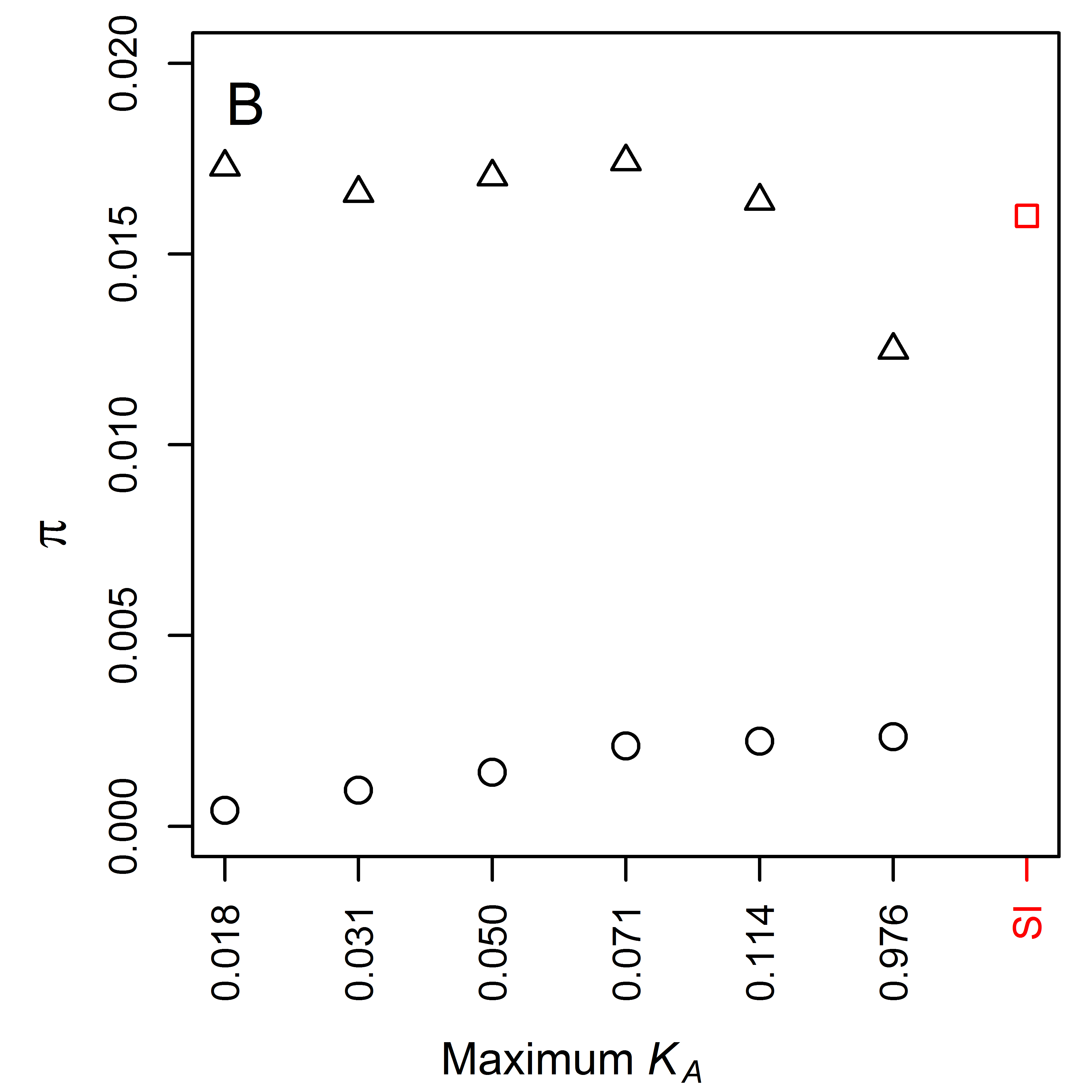 |
| --- | --- |
| 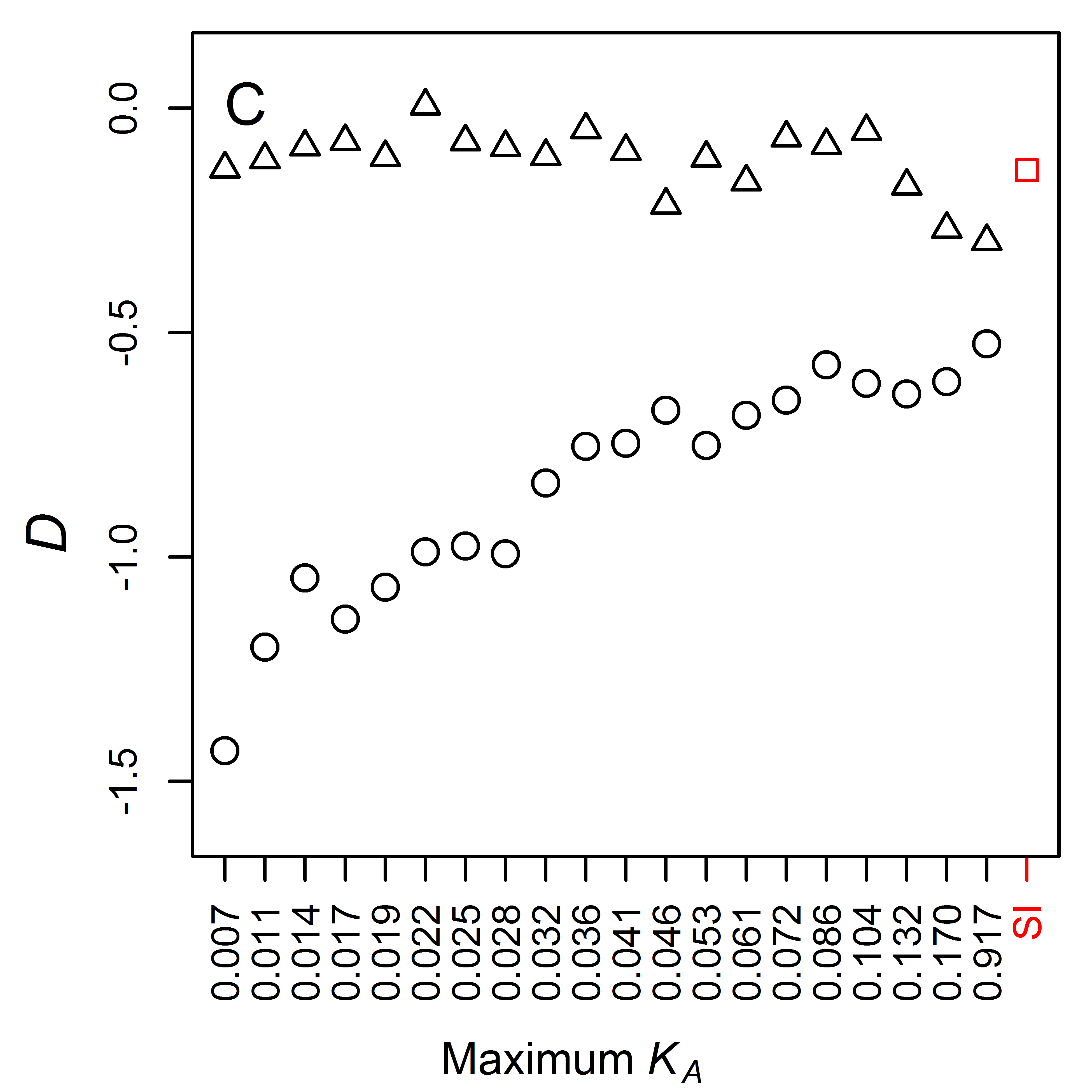 | 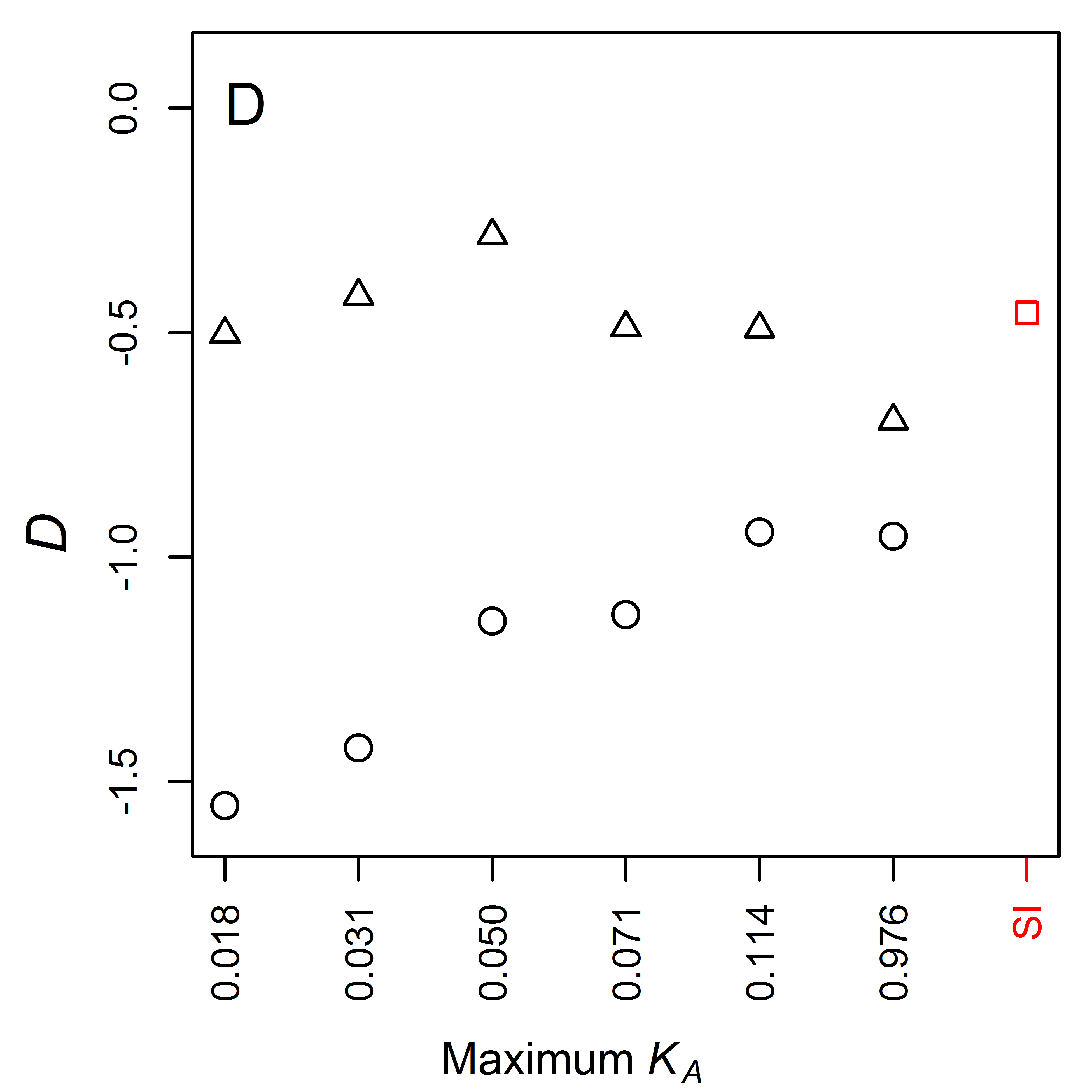 |

**Figure 2**.

| 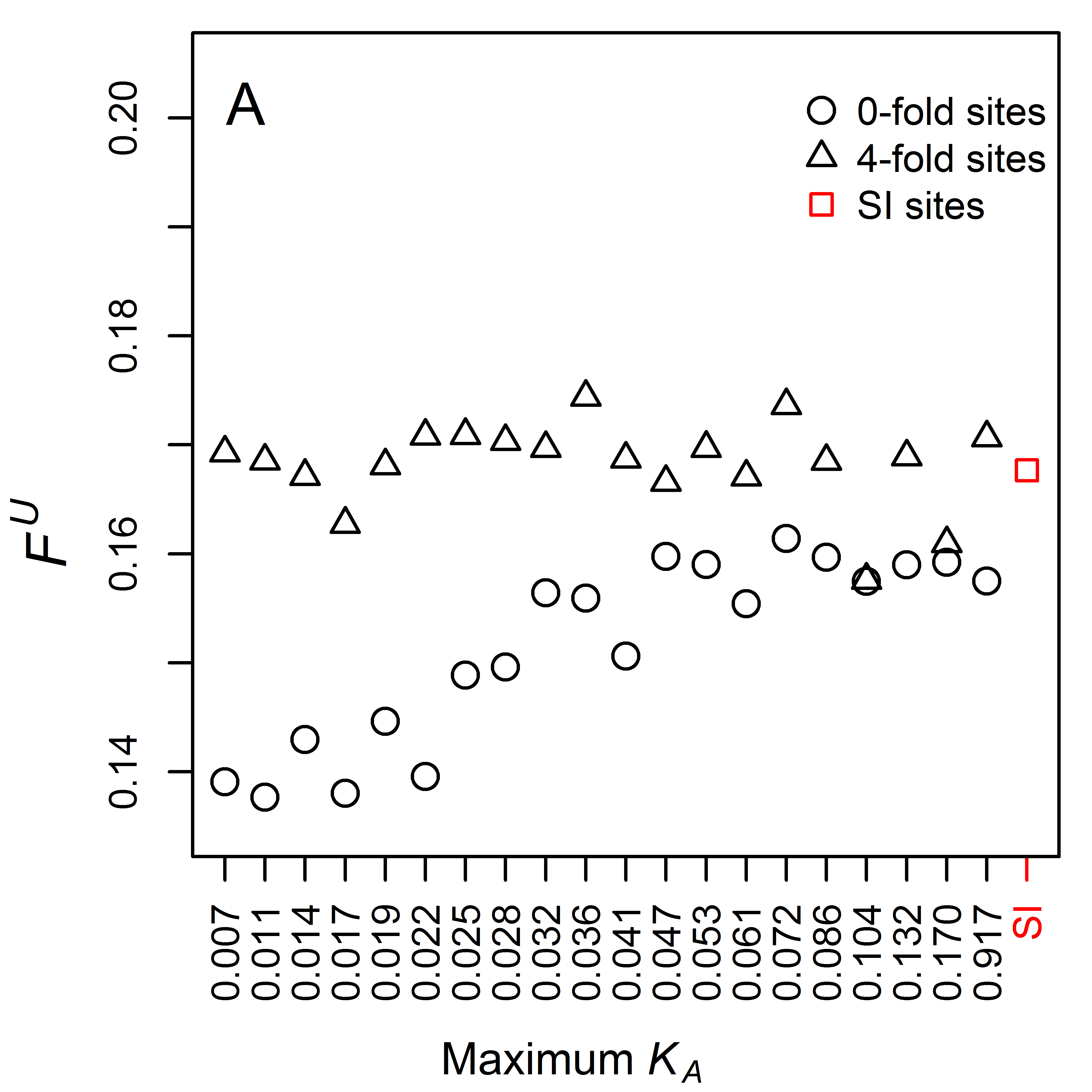 | 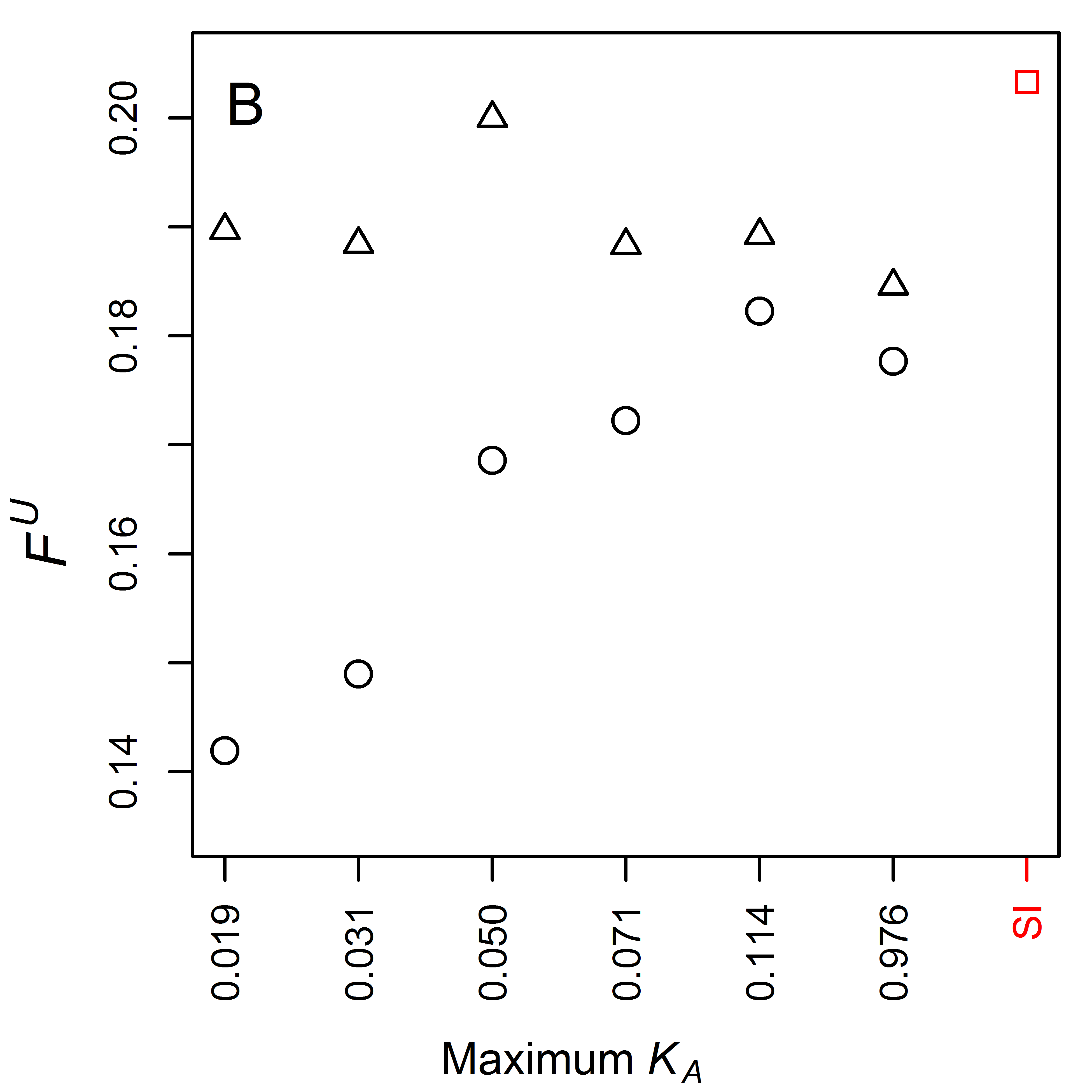 |
| --- | --- |
| 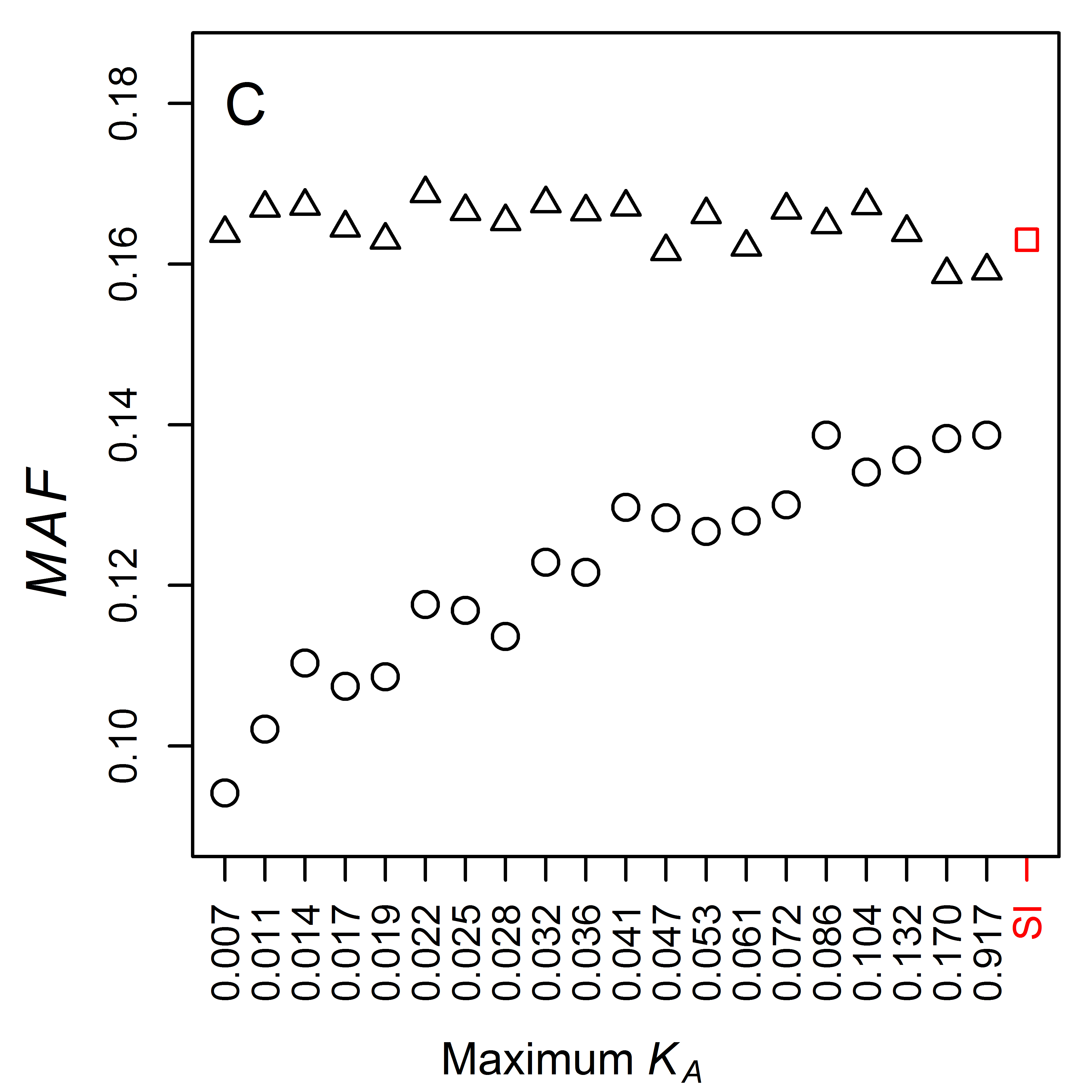 | 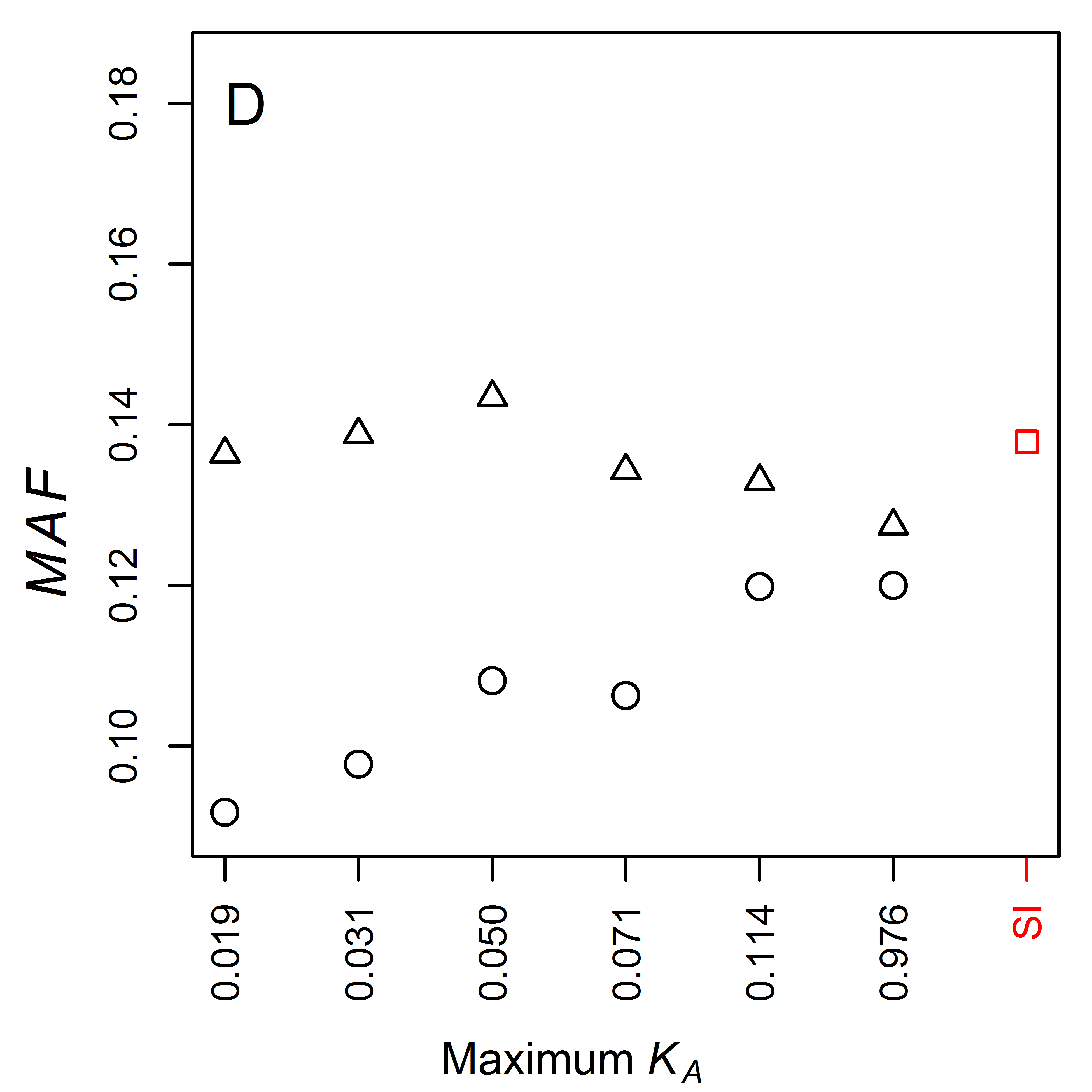 |
| 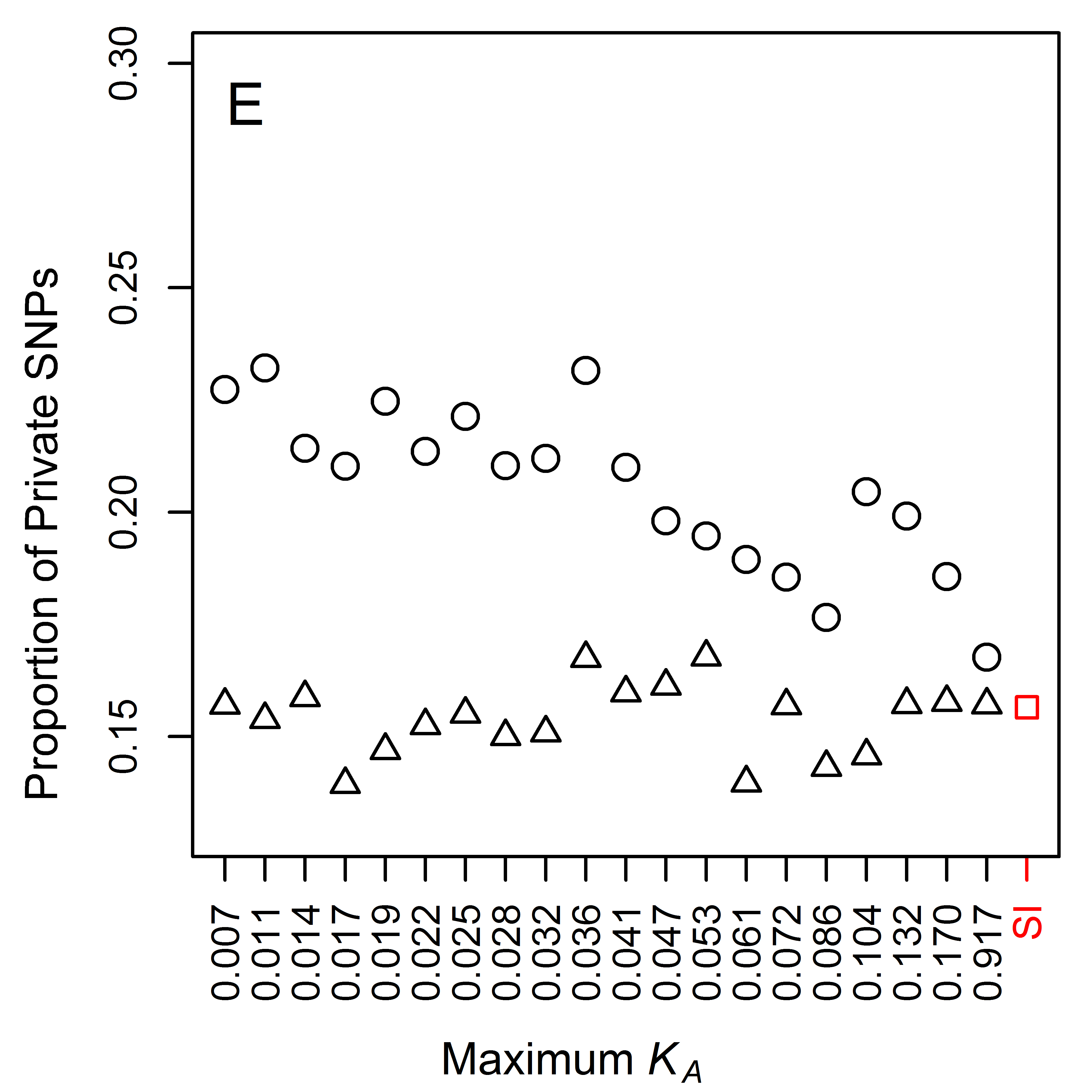 | 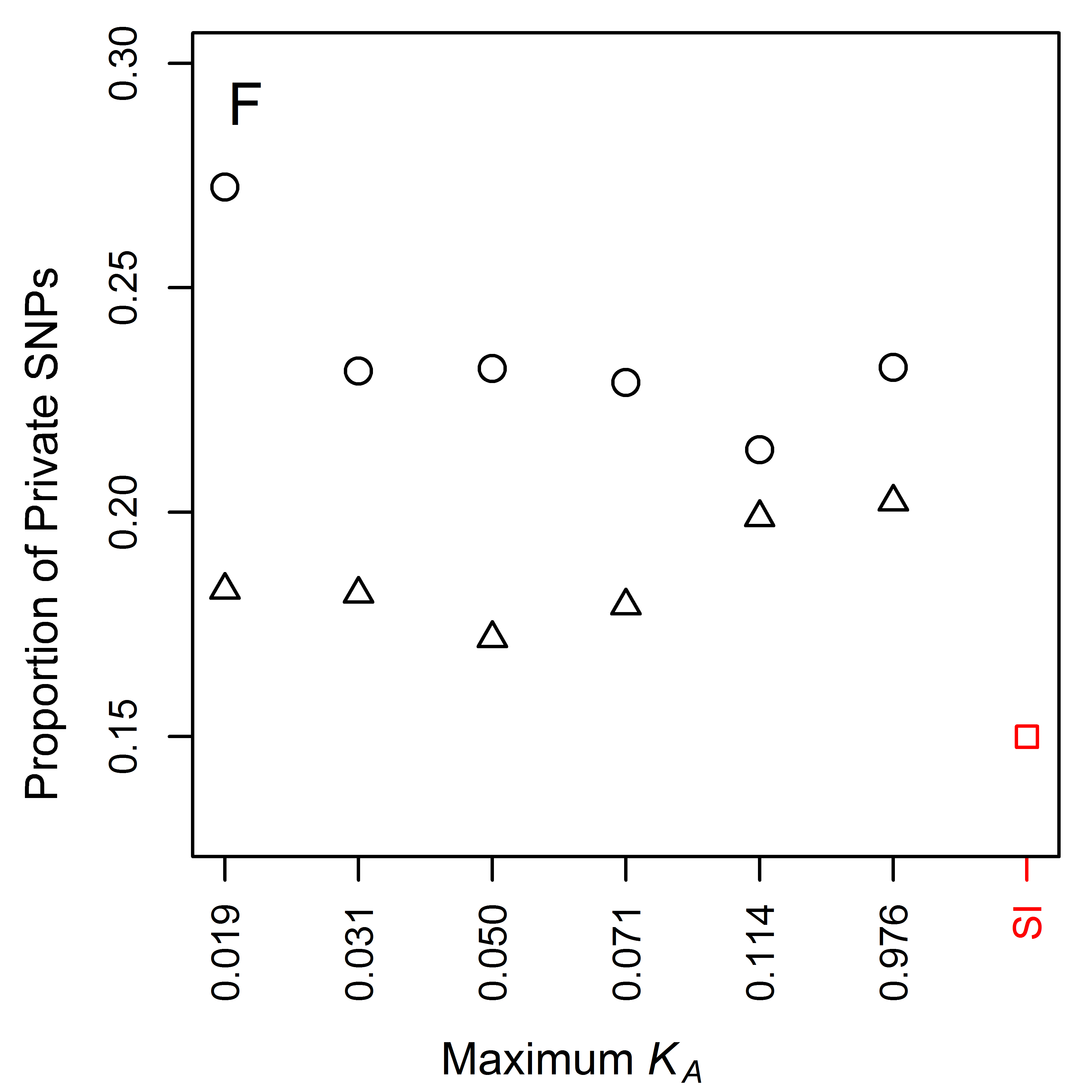 |

**Figure 3**.

| 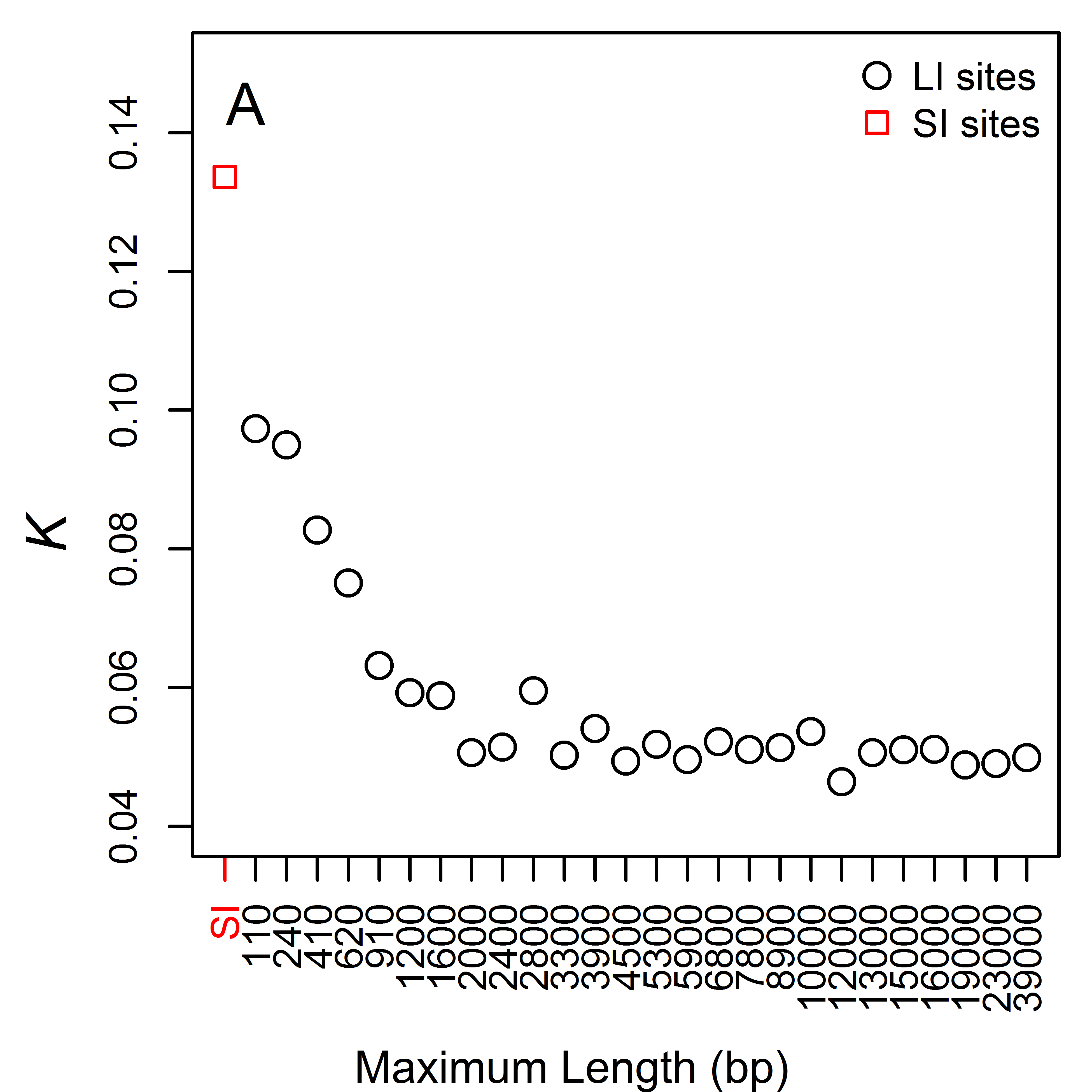 | 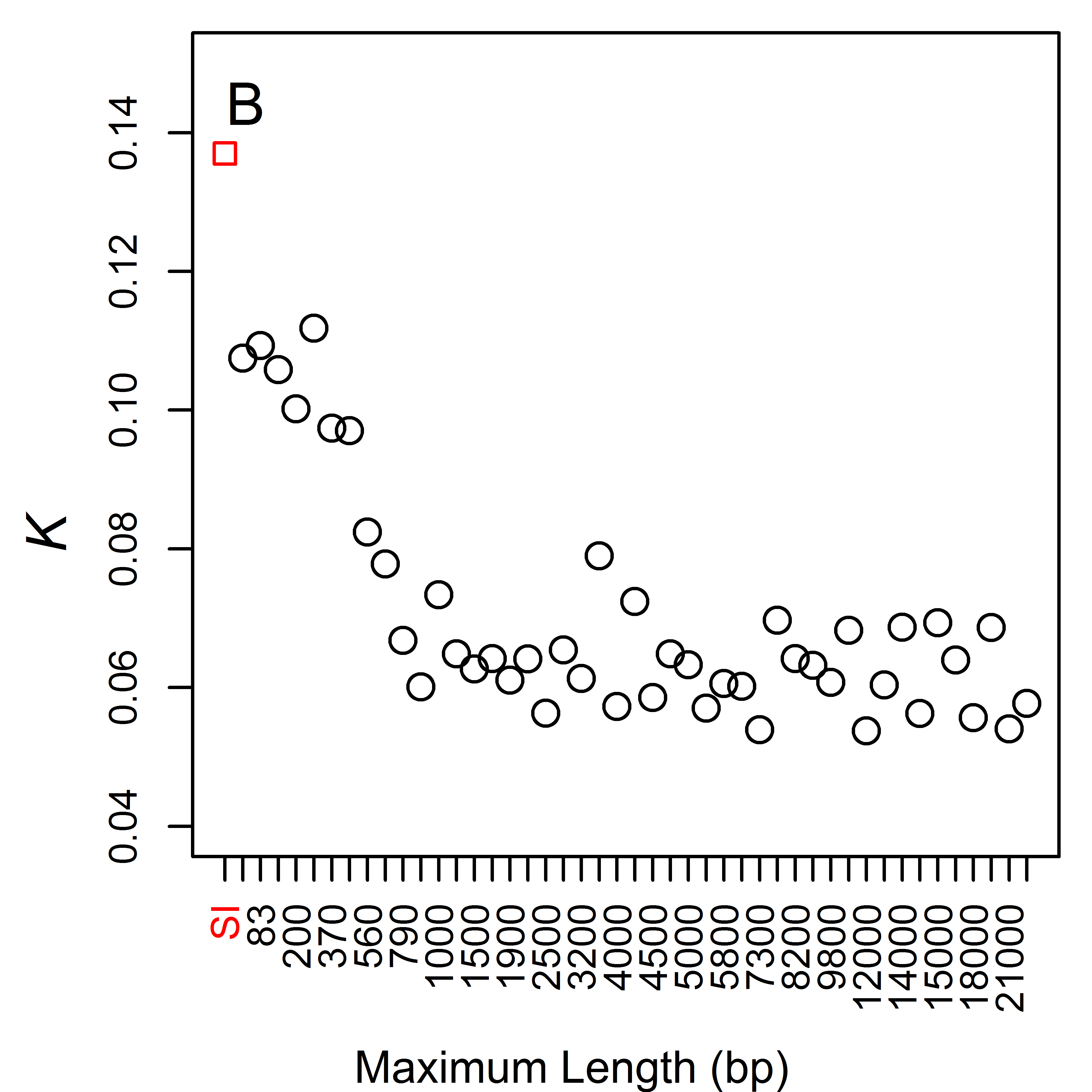 |
| --- | --- |
| 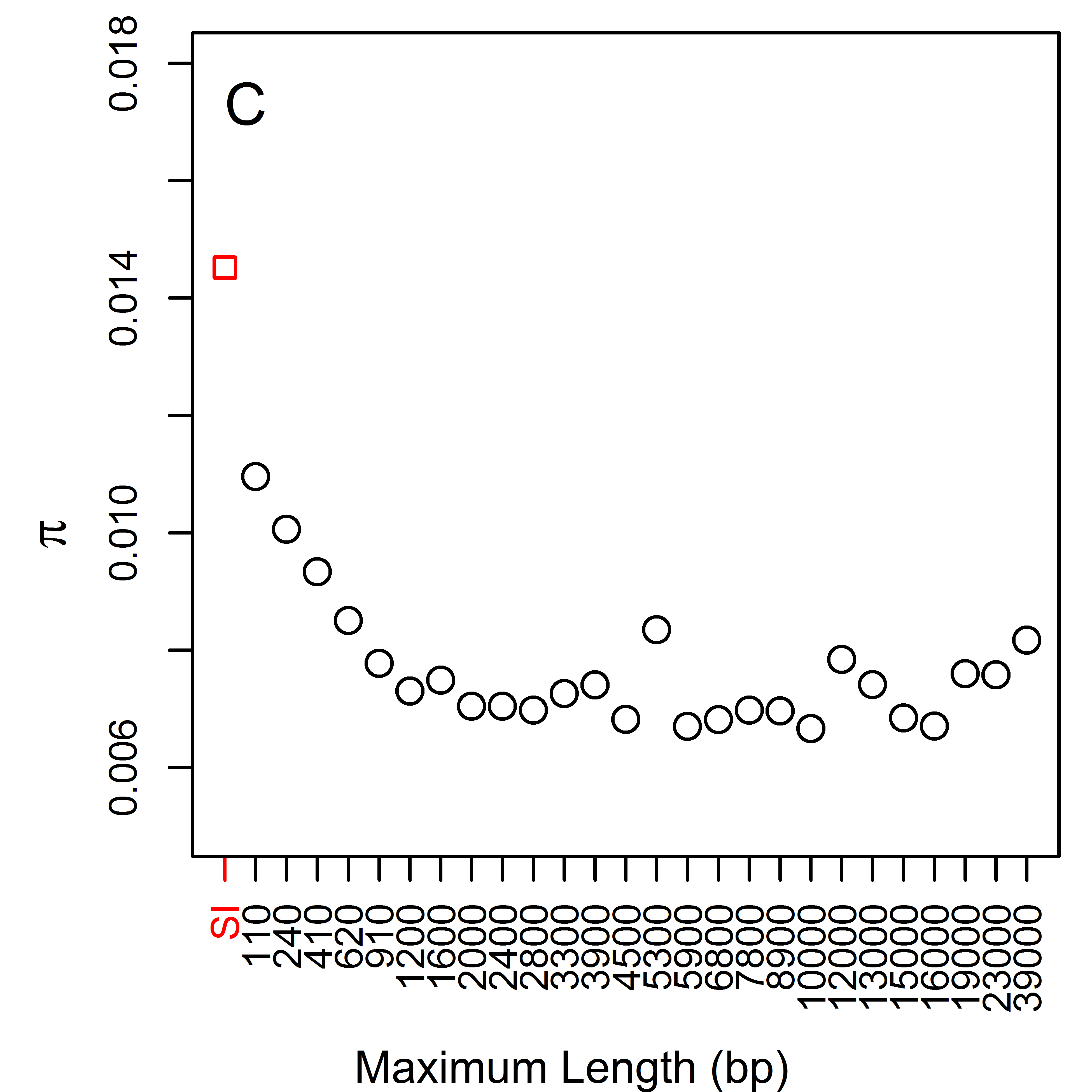 | 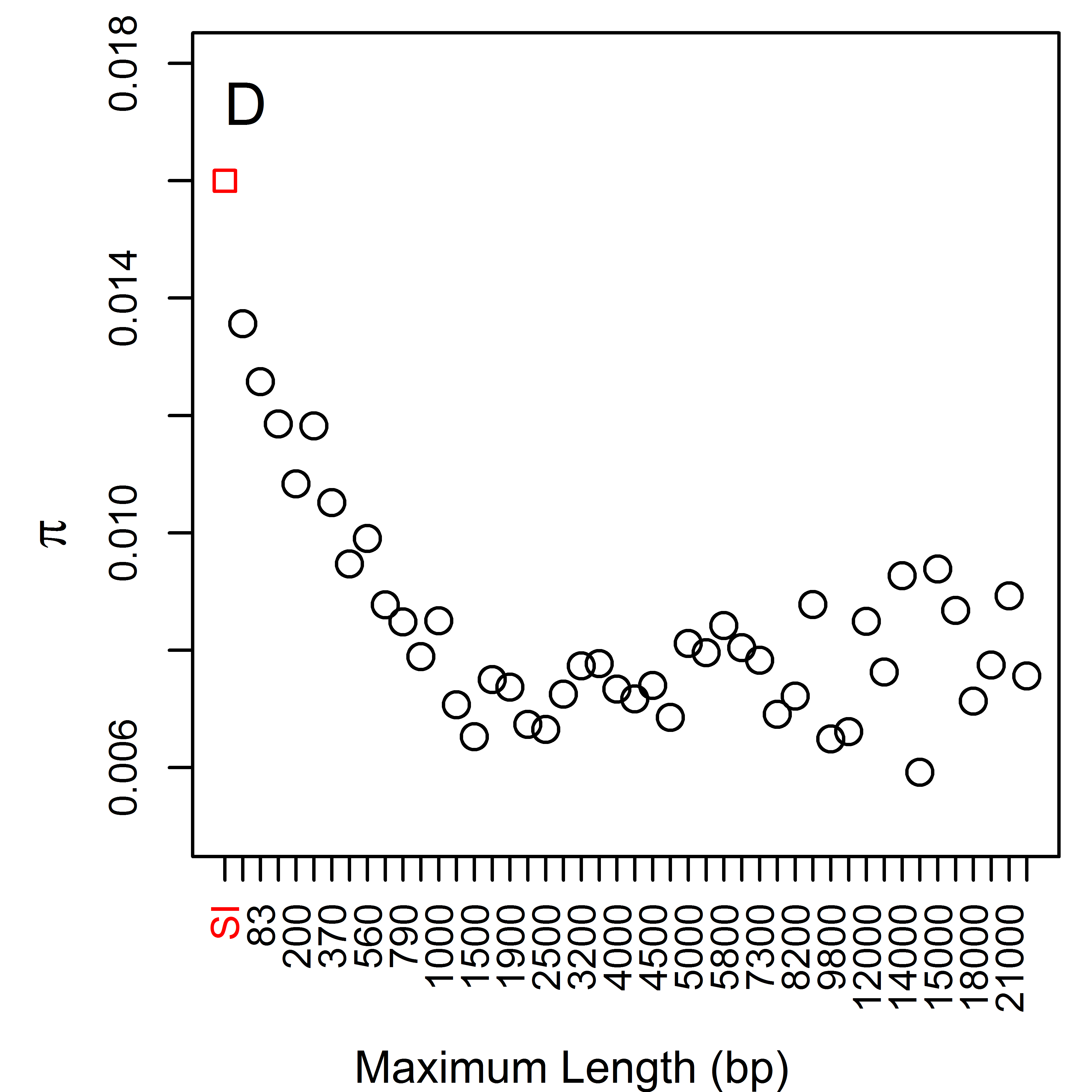 |
| 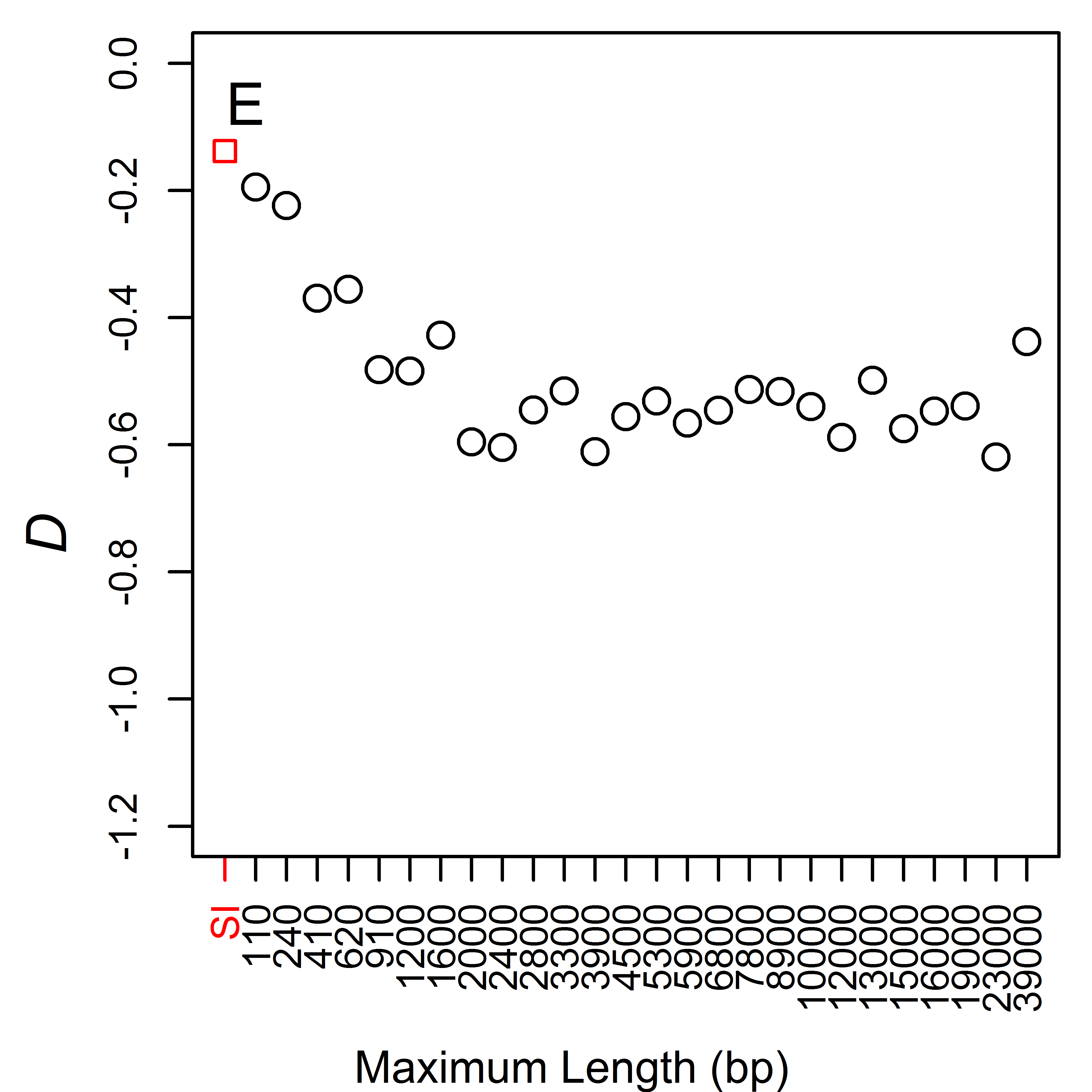 | 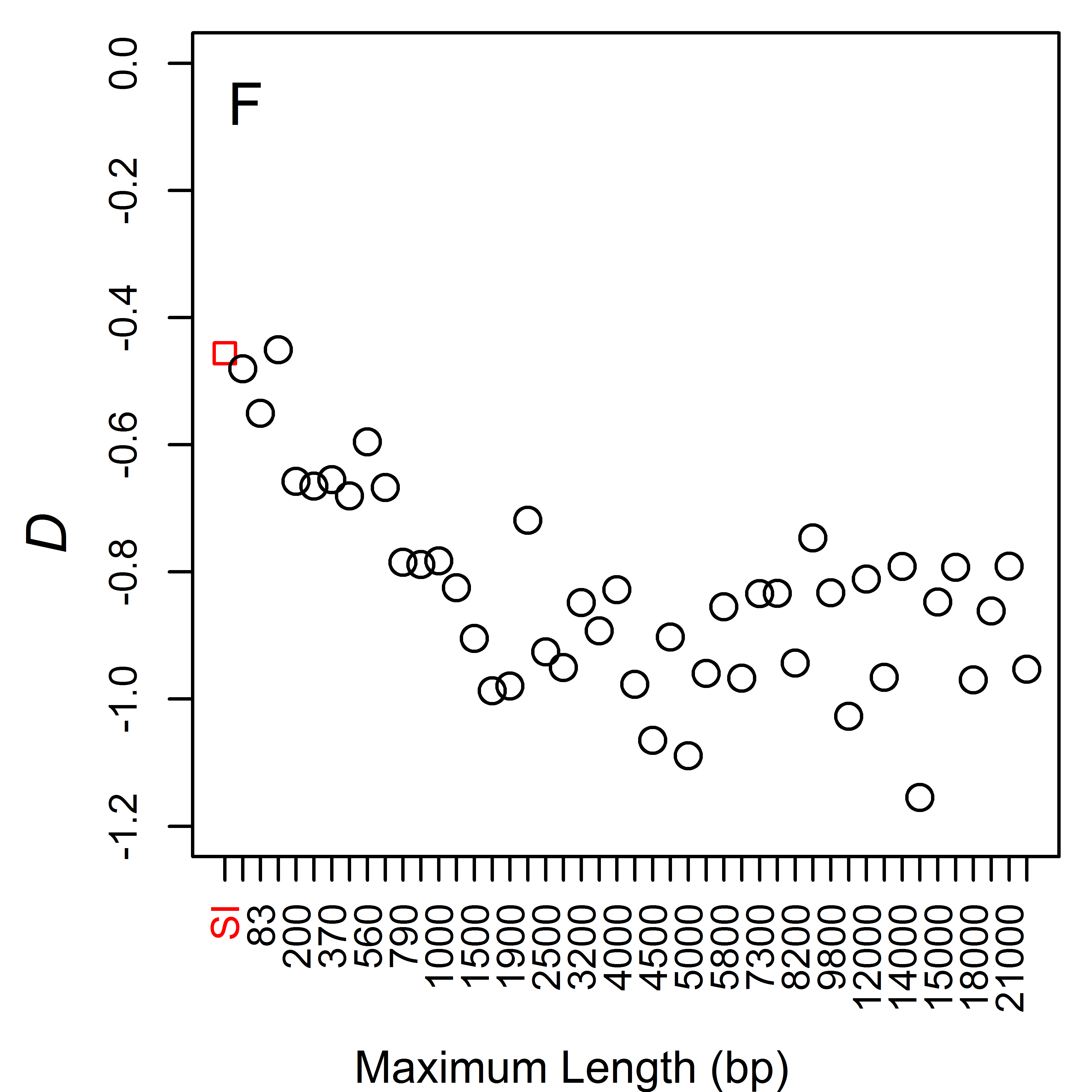 |

**Figure 4**.

| 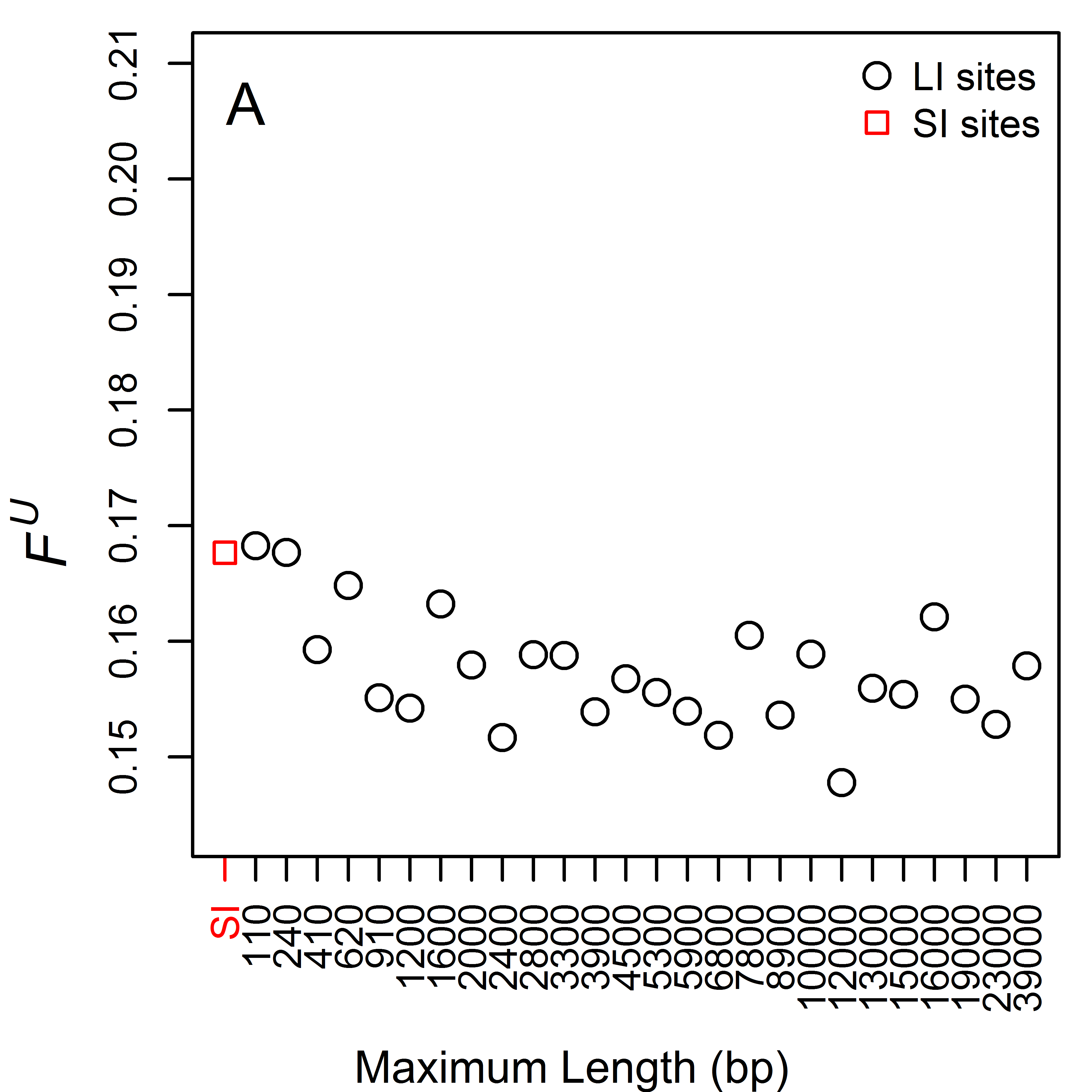 | 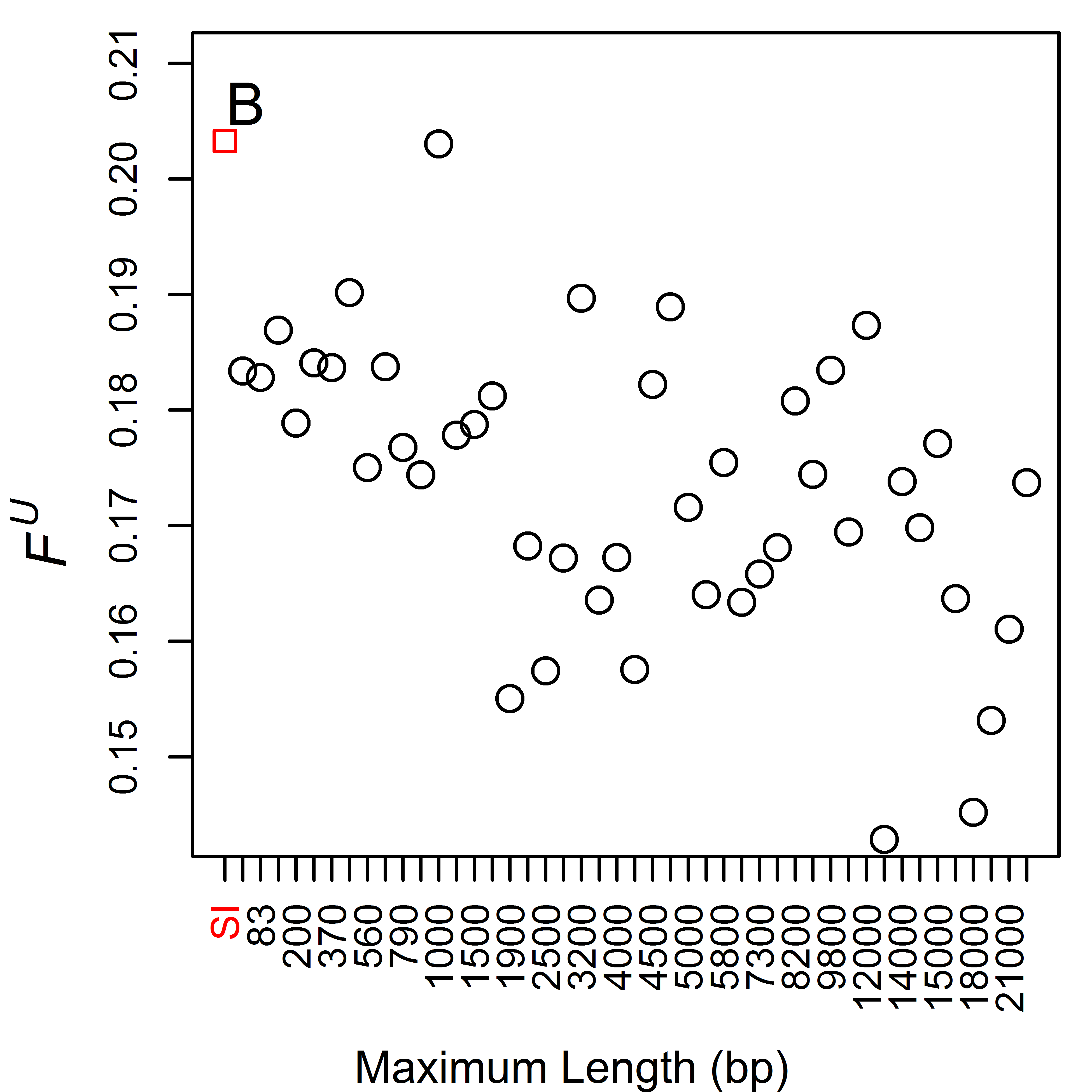 |
| --- | --- |
|  |  |

**Figure 5**.

| 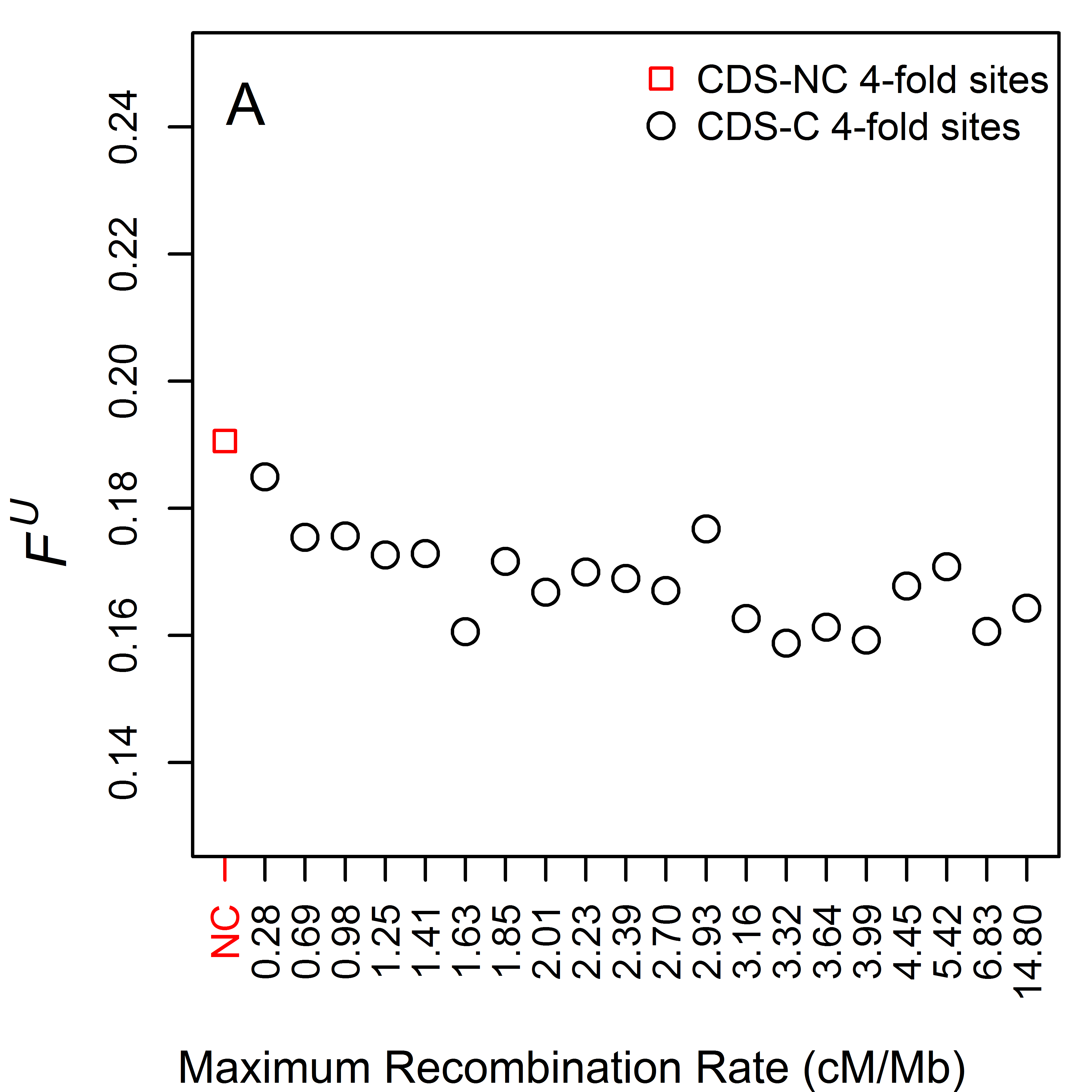 | 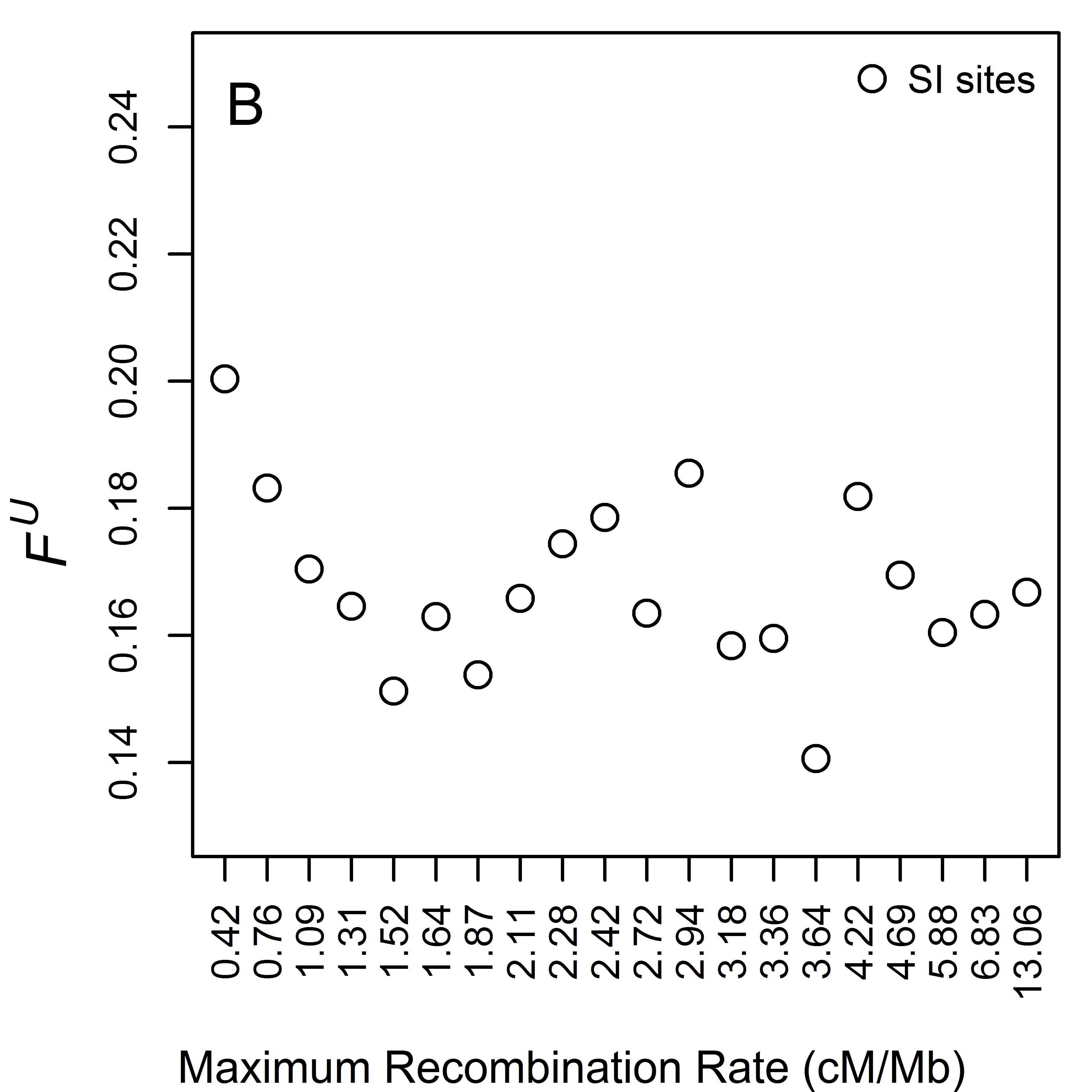 |
| --- | --- |
|  |  |
